# Enzymatic Interesterification of Cold-Pressed Maqui (*Aristotelia chilensis* (Mol.) Stuntz) Seed Oil and Belly Oil from Rainbow Trout (*Oncorhynchus mykiss*) Through Supercritical CO_2_

**DOI:** 10.3390/md22120547

**Published:** 2024-12-04

**Authors:** Francisca Reinoso, Alicia Rodríguez, Camila Sánchez, Benjamín Claria, Nalda Romero, Alejandra Espinosa, María Elsa Pando, Rodrigo Valenzuela, Dayana Apaza, Gretel Dovale-Rosabal, Santiago P. Aubourg

**Affiliations:** 1 Department of Food Science and Chemical Technology, Faculty of Chemical and Pharmaceutical Sciences, University of Chile, Dr. Carlos Lorca Tobar 964, Santiago 8380494, Chile; francisca.reinoso@ug.uchile.cl (F.R.); camila.sanchez.s@ug.uchile.cl (C.S.); benjamin.claria@ug.uchile.cl (B.C.); nromero@uchile.cl (N.R.); dayana.apaza@ug.uchile.cl (D.A.); gretel.dovale@ug.uchile.cl (G.D.-R.); 2 Department of Medical Technology, Faculty of Medicine, University of Chile, Independencia 1027, Santiago 8380000, Chile; ealejand@uchile.cl; 3 Department of Nutrition, Faculty of Medicine, University of Chile, Independencia 1027, Santiago 8380000, Chile; pandosanmartin@uchile.cl (M.E.P.); rvalenzuelab@uchile.cl (R.V.); 4 Department of Food Technology, Marine Research Institute (CSIC), Eduardo Cabello 6, 36208 Vigo, Spain

**Keywords:** cold-pressed maqui seed oil, *Aristotelia chilensis* (Mol.) Stuntz, belly oil, rainbow trout, supercritical CO_2_, enzymatic interesterification, RSM optimization, EPA content, DHA content, tocopherols

## Abstract

A new antioxidant lipid (AL) was synthesized from rainbow trout (*Oncorhynchus mykiss*) belly oil and cold-pressed maqui (CPM) (*Aristotelia chilensis* (Mol.) Stuntz) seed oil via enzymatic interesterification using *Thermomyces lanuginosus* in supercritical CO_2_ medium. A Box–Behnken design with 15 experiments was employed, with the independent variables being the following: belly oil/CPM oil ratio (10/90, 50/50, and 90/10, *w*/*w*), supercritical CO_2_ temperature (40.0, 50.0, and 60.0 °C), and supercritical CO_2_ pressure (100.0, 200.0, and 300.0 bar) for enzymatic interesterification. A multiple optimization was conducted based on the response variables yield and eicosapentaenoic acid (EPA), docosahexaenoic acid (DHA), and tocopherol contents. The optimized conditions for the AL synthesis were: 81.4/18.6 (*w*/*w*), 40.0 °C and 299.99 bar, respectively. The corresponding responses variables were: 77.10% for yield, 5.12 and 4.95 g·100 g^−1^ total fatty acids for EPA and DHA, respectively, and 217.96, 4.28, 3.48, 64.48, and 6.39 mg·kg^−1^ oil for α-tocopherol, α-tocotrienol, β-tocopherol, γ-tocopherol, and δ-tocopherol, respectively. A novel AL was successfully synthesized starting from two abundant natural resources commonly considered as by-products during industrial processing. In agreement with the high EPA, DHA, and tocopherol presence, this AL can be recommended to be employed in nutritional and therapeutic supplements, according to its health benefits, particularly concerning antioxidant and anti-inflammatory properties.

## 1. Introduction

Lipids are defined as hydrophobic or amphipathic molecules and can be totally or partially generated through condensations of thioesters or units of isoprene [1]. They are considered key nutrients that influence the early growth and development of human beings. Likewise, lipids contribute to the prevention and control of chronic non-transmissible diseases like obesity, high blood pressure, cardiovascular health, among others [2]. Fahy et al. [1] divide lipids into eight categories (fatty acyls, glycerolipids, glycerophospholipids, sphingolipids, sterol lipids, prenol lipids, saccharolipids, and polyketides). Free fatty acids (FFAs) are the basic lipid constituents and play a role as an energy source to perform the metabolic and/or structural functions required by organisms. Eicosapentaenoic acid (EPA) (C20:5n-3) and docosahexaenoic acid (DHA) (C22:6n-3) are two of the most important omega-3 long-chain polyunsaturated fatty acids (n-3 LC-PUFAs) in human nutrition. Both EPA and DHA are primarily found in seafood including fish, shellfish, and other marine organisms, while DHA is also present in algae [3,4,5].

Cohort studies of EPA and DHA intakes have demonstrated physiological benefits on blood pressure, inflammation processes, endothelial function, cardiac diastolic function, and consistent evidence for a reduced risk of fatal CHD and sudden cardiac death [6,7,8,9]. Furthermore, there is significant evidence indicating that DHA plays a crucial role in brain and retinal development during fetal development and the first two years of life [6,10,11,12].

According to Rimm et al. [4], it is recommended to consume non-fried seafood, especially species higher in n-3 LCPUFA. Therefore, consumption 1- to 2-times per week would be favorable for cardiovascular benefits, including reduced risk of cardiac death, CHD, and ischemic stroke. This recommendation is consistent with the AHA 2020 Impact Goals, which includes seafood as part of the healthy dietary pattern goals, and the 2015 American Heart Association (AHA) Diet and Lifestyle. In the 2015–2020 period, Dietary Guidelines for Americans and the Associated 2015 Dietary Guidelines Advisory Committee Report indicated that 8 oz of seafood or 2 servings per week would be sustainable and environmentally friendly.

Rainbow trout (*Oncorhynchus mykiss*) has received great attention due to wide aquaculture production in many countries. Previous research has shown a high yield of n-3 LCPUFA content in this species [13,14]. Trimming fish involves the process of removing unwanted parts to enhance the quality and presentation of the fish. Commonly, this process includes removing scales, cleaning the gut, cutting off fins and tail, and removing skin and belly. The belly muscle corresponds to the longitudinal cut of the fish, obtained from the central part of the stomach from which the skin, bones, and stirrup have not been removed [15]. Rainbow trout belly is a by-product resulting from the trimming process for export of rainbow trout filet. The extraction of oil from the belly of rainbow trout can be obtained using the modified Radin method [16,17,18,19]. Previous research found that the n-3 long-chain PUFA content in the belly oil of rainbow trout was 28.31 (g·100 g^−1^ total FAs), with EPA + DHA comprising 19.93 (g·100 g^−1^ total FAs) [19].

Since lipids of marine species are highly unsaturated, contact with oxygen, especially in the presence of light or other catalysts, can lead to a wide range of lipid oxidation compounds (peroxides, conjugated dienes, carbonyls, etc.) and the loss of some essential and beneficial free fatty acids (FFAs) for the human diet [4,20]. Lipid oxidation compounds can interact with protein derivatives, leading to a nutritional loss of the product [21,22]. To avoid lipid oxidation, the use of antioxidants has been shown to be effective to minimize or prevent this damage pathway in seafood, thus, retarding the formation of toxic products and maintaining the nutritional quality [23,24]. In agreement with safety concerns, great efforts are being made nowadays to replace synthetic antioxidants with others obtained from natural resources such as terrestrial plants and algae [25,26]. The high number of double bonds present in EPA and DHA makes them more susceptible to rapid oxidation, leading to deterioration of quality [27]. The natural antioxidants used in foodstuffs to protect fats against oxidative rancidity, such as α-tocopherol contained in vegetable oil, have long been used for food processing. Antioxidants may act at different levels in the oxidative process, e.g., by (1) scavenging initiating radicals, (2) binding to metal ions, (3) scavenging peroxyl radicals, or (4) removing oxidized-damaged biomolecules, and other kinds of action [28].

It has been reported that α-, β-, γ-, and δ-tocopherol, tocotrienols, carotenes, and sterols are present in maqui seed oil, all of them with potential antioxidant properties [29]. Maqui is an endemic tree species widely distributed in Chile and Argentina [30]. Industrialization of maqui mainly involves obtaining juice and extracts from the pulp, generating a by-product with a big proportion of maqui seed (50%), which could be a good source of nutrients [29]. Wild maqui fruit has the highest antioxidant capacity of any marketed fruit, and has been used for food, medicinal properties, pharmaceuticals, and cosmetics products [29,30,31,32,33]. The interest in maqui is because of its health effects attributed mainly to the high content of polyphenols and its composition [34], among them, prevention of endothelial dysfunction and cardiovascular disease [29,35,36]. Moreover, the following have also been associated: inhibition of lipid accumulation, reduction in plasma cholesterol levels, and improved nitric oxide bioavailability [37]. Regarding seed oil from maqui berry, Bastías-Montes et al. [29] have reported the presence of all tocopherols and tocotrienols compounds; additionally, β-sitosterol was the only sterol found in higher amounts than other vegetable oils [29]. Sterols, such as ergosterol, β-sitosterol, stigmasterol and campesterol, present anti-inflammatory effects and the ability to block cholesterol absorption [38,39]. In fact, maqui seed oil has already been reported to have a higher antioxidant capacity than olive oil or canola oil [29]. Meanwhile, α-tocopherol exhibits anti-inflammatory activity and modulates the expression of proteins involved in the cholesterol metabolism [40,41], and it protects PUFAs from membranes and other cellular structures from lipid peroxidation. Furthermore, γ-tocopherol is considered as the most potent free radical scavenger among vitamin E compounds. In addition, it has a strong anti-inflammatory activity and is related to the inhibition of carcinogenesis [42,43].

Due to the above-mentioned factors, there has been a significant demand for beneficial products for health and, therefore, an increased interest in structured phenolic lipids due to their probable antioxidant and anti-inflammatory properties, among others [23,24]. These structured phenolic lipids can be obtained through interesterification [25]. Interesterification processes occur when FFAs are exchanged with each other [44]. Enzymatic interesterification is defined as the ester exchange or ester–ester exchange that can occur between two monoesters, or a mono- and a polyester, or two polyesters. Reactions between two or more polyesters are the most important. Enzymatic interesterification is used to modify oil and fat by enzymes to obtain improved structured lipids with the desired technological properties [45,46]. The enzymatic interesterification of EPA/DHA-rich antioxidant lipid that includes an EPA or DHA, phenols, α-, β-tocopherol compounds, and tocotrienols from cold pressed maqui seed oil, could potentially lead to an improved anti-inflammatory response by obtaining an EPA/DHA-rich biologically active antioxidant lipid. A lipase is used as a catalyst instead of a chemical catalyst. Enzymes are natural products that operate at relatively low temperatures in reactions (40–60 °C). Lipases as catalysts allow FFAs to rearrange themselves in the glycerol molecule in certain positions during the process. The immobilized lipase B from TL IM (*Thermomyces lanuginosus*) is a specific lipase for hydrolysis in the sn-1,3 positions. The TL IM lipase selects the acyl group of the sn-1,3 position of the glycerol skeleton, also having the ability to select certain substrates and their stereoisomers being a regioselective enzyme [47]. TL IM lipase has been shown to be specific in the enzymatic interesterification of TG, meaning that acyl groups are introduced in specific positions of the glycerol since the reaction occurs randomly with respect to position. These novel molecules could offer numerous beneficial properties of both n-3 LCPUFA and natural antioxidant compounds of cold-pressed maqui seed oil [48,49,50].

Supercritical CO_2_ is particularly advantageous for enzymatic catalysis in enzymatic interesterification reactions, as it maintains enzyme stability by allowing reactions to occur at optimal temperatures without significant denaturation [51,52]. The applications of supercritical CO_2_ are drawing growing attention due to the non-toxic properties, carbon capture potential, and industrial scalability. Supercritical CO_2_ is achieved when CO_2_ is compressed to a pressure of 73.9 bar and a temperature of 31.1 °C, above its critical point. In these conditions, CO_2_ does not liquefy but reaches a dense gaseous state, behaving like a solvent [53,54]. Supercritical CO_2_ is a released gas that can be purchased at a low price. It can replace other organic solvents used in industry, thereby preventing the release of toxic substances into the environment [51]. This method also contributes to the reduction in the carbon footprint. As a non-toxic solvent, it finds applications in various fields such as pharmaceuticals [52]. Supercritical CO_2_ has both gas-like and liquid-like properties, including low surface tension, high diffusivity, and low viscosity, which lead to its excellent dissolving ability and high mass transfer rate [53,54]. The use of supercritical CO_2_ for the synthesis of biologically active lipids significantly minimizes the reliance on organic solvents, avoids waste disposal problems and the need for potentially toxic and flammable solvents, and reduces reaction times [51,54]. Among supercritical fluids, CO_2_, is found to be a convenient solvent for food applications [52,53,54,55,56].

With the aim of studying the effect of the different variables in the enzymatic interesterification process under supercritical CO_2_ conditions, an experimental design using Response Surface Methodology (RSM) was performed in order to enhance efficiency and minimize time in experimental investigation [19,57]. In this work, a new antioxidant lipid (AL) was obtained from rainbow trout (*Oncorhynchus mykiss*) belly oil with cold-pressed maqui (*Aristotelia chilensis* (Mol.) Stuntz) seed oil through enzymatic interesterification in supercritical CO_2_ medium.

## 2. Results

### 2.1. Extraction Yield of Belly Oil from Rainbow Trout

The extraction was carried out as described in Section 4.2. A total of five extractions were performed, yielding an average value of 37.6 ± 7.5%. The frozen belly pieces used were six pieces that weighed an average value of 102.1 ± 2.9 g.

### 2.2. Enzymatic Interesterification Under Supercritical CO_2_ of Belly Oil from Rainbow Trout and Cold-Pressed Maqui Seed Oil Using the Experimental Design by RSM

Enzymatic interesterification under supercritical CO_2_ was carried out using a Box–Behnken experimental design with Response Surface Methodology (RSM). A total of fifteen enzymatically interesterified experiments were conducted. The independent variables measured were the belly oil from rainbow trout/cold-pressed maqui seed oil ratio and supercritical CO_2_ temperature and pressure, while the response variables measured were yield and EPA, DHA, and tocopherol contents.

#### 2.2.1. Effect of Independent Variables of the Enzymatic Interesterification Process on Yield (%)

Table 1 presents the effect of the belly oil from rainbow trout/cold-pressed maqui seed oil ratio (*w*/*w*) and supercritical CO_2_ temperature (°C) and pressure (bar) as independent variables of the enzymatic interesterification process of belly oil from rainbow trout and cold-pressed maqui seed oil on yield (%). Experiments No. 11, 12, and 8 achieved the highest yields, i.e., 71.0%, 64.0%, and 63.0%, respectively. Conversely, experiments No. 9, 5, and 6 led to the lowest yields, i.e., 3%, 1%, and 1%, respectively; in such cases, the oil extraction was negligible so that no further analysis could be performed.

**Figure 1:**
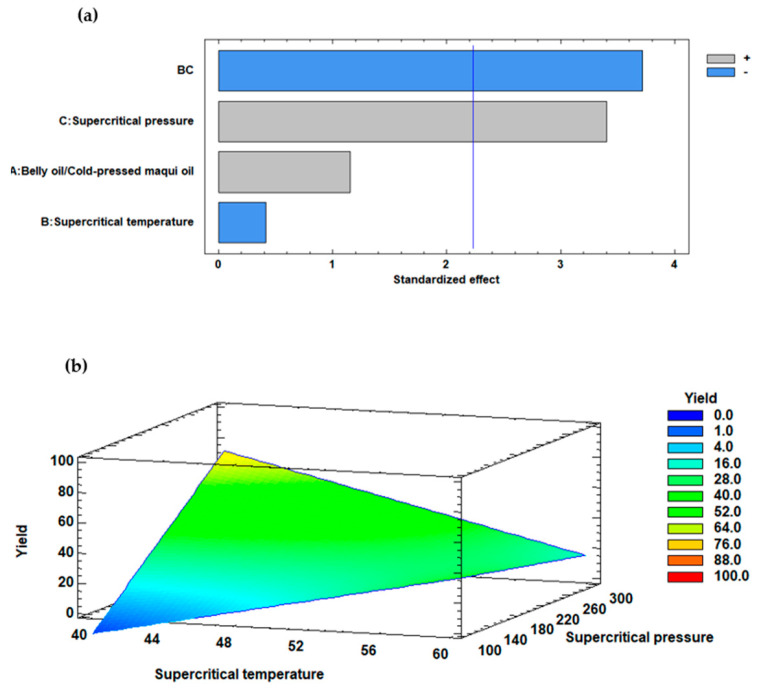
Effect of independent variables of the interesterification process on yield (%). (**a**) Standardized Pareto diagram for the response variable yield (%). A higher value than the blue line mark indicates a significant effect, *p* < 0.05. (**b**) Estimated response surface. Diagram for the response variable yield (%) of supercritical CO_2_ temperature (°C) vs. supercritical CO_2_ pressure (bar) at constant belly from rainbow trout/cold-pressed maqui seed oil (*w*/*w*) ratio.

The results in Table 1 can be explained by the behavior shown in Figure 1a, which displays the standardized Pareto diagram for yield. This figure illustrates the independent variables that influenced the yield outcome. The supercritical CO_2_ pressure variable (C) had a significant positive impact, as the bar representing it surpassed the blue vertical line, with a *p*-value < 0.05, whereas the interaction of the supercritical CO_2_ temperature and pressure variable (BC) presented a negative effect on yield (*p*-value < 0.05). Figure 1b presents the estimated response surface diagram, which shows that increasing the supercritical CO_2_ pressure variable (C) and decreasing the supercritical CO_2_ temperature improves the yield.

#### 2.2.2. Effect of Independent Variables of the Interesterification Process on EPA and DHA Content

Table 2 shows that the highest EPA and DHA values were obtained in experiments No. 8, 4, and 2, which contained the highest proportion of belly oil from rainbow trout/cold-pressed maqui seed oil ratio of 90/10 (*w*/*w*), according to Table 1.

**Figure 2:**
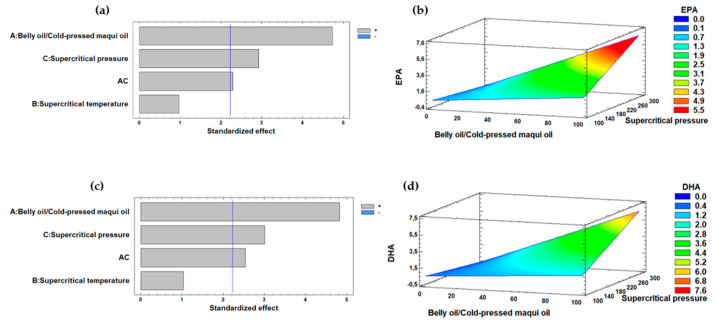
Effect of independent variables of the interesterification process on EPA and DHA (mg·kg^−1^ oil). Panels (**a**,**c**): Standardized Pareto diagram for the response variable EPA and DHA (mg·kg^−1^ oil), respectively. A higher value than the blue line mark indicates a significant effect, *p* < 0.05. Panels (**b**,**d**): Estimated response surface diagram for the response variable EPA and DHA (mg·kg^−1^ oil). Belly oil from rainbow trout/cold-pressed maqui seed oil (*w*/*w*) relation vs. supercritical CO_2_ pressure (bar) at constant supercritical CO_2_ temperature (°C).

Figure 2 illustrates the effect of the independent variables of the interesterification process on the EPA and DHA content. For it, the standardized Pareto diagrams for EPA and DHA content and estimated response surfaces for the response variables EPA and DHA content are depicted. The diagrams reveal a similar behavior of the independent variables for both FAs. Both EPA and DHA were significantly influenced positively, with the belly oil from rainbow trout/cold-pressed maqui seed oil (*w*/*w*) ratio having the greatest impact, followed by supercritical CO_2_ pressure. Figure 2b,d shows the estimated response surfaces, indicating that increasing the belly oil from rainbow trout/cold-pressed maqui seed oil (*w*/*w*) ratio and supercritical CO_2_ pressure enhanced the concentration of EPA and DHA in the final product. At supercritical CO_2_ pressures of 100.0 bar, the content of EPA and DHA was negligible.

#### 2.2.3. Effect of Independent Variables of the Interesterification Process on Tocopherol Compound Values

The results obtained from the interesterification process experiments for tocopherols are shown in Table 3. Five tocopherols were identified and quantified: α-, β-, γ-, δ-tocopherol, and α-tocotrienol (mg·kg^−1^ oil).

Table 3 shows that α-tocopherol had the highest concentration at 256.09 ± 10.86 ^b^ (mg·kg^−1^ oil) in experiment No. 14; this experiment corresponds to an enzymatic interesterification process with a 50/50 (*w*/*w*) belly oil from rainbow trout/cold-pressed maqui seed oil ratio, 50.0 °C supercritical CO_2_ temperature, and 200.0 bar supercritical CO_2_ pressure (Table 1). The second highest concentration was observed for γ-tocopherol (154.51 ± 12.15 mg·kg^−1^ oil) in experiment No. 7, with a 10/9 (*w*/*w*) belly oil from rainbow trout/cold-pressed maqui seed oil ratio, 50.0 °C supercritical CO_2_ temperature, and 300 bar supercritical CO_2_ pressure (Table 1).

**Figure 3:**
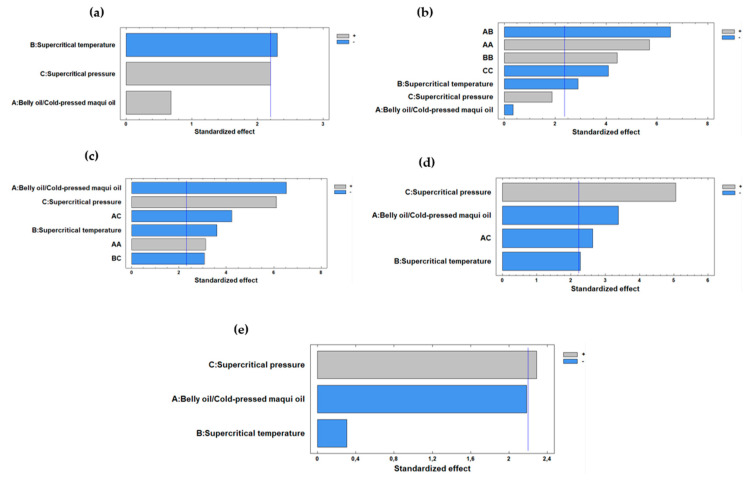
Standardized Pareto diagrams for the response variable tocopherols: (**a**) α-tocopherol, (**b**) α-tocotrienol, (**c**) β-tocopherol, (**d**) γ-tocopherol, and (**e**) δ-tocopherol. A higher value than the blue line mark indicates a significant effect, *p* < 0.05.

Figure 3 displays the Pareto diagrams for the response variable tocopherols, showing that supercritical CO_2_ pressure was the independent variable with the most positive effect on tocopherol values overall (Figure 3a,c,d,e). In contrast, supercritical CO_2_ temperature was the independent variable that generally had the most negative effect (Figure 3a–d) (*p*-value < 0.05). Meanwhile, the ratio of belly oil from rainbow trout to cold-pressed maqui seed oil was the independent variable that negatively affected β-tocopherol, γ-tocopherol, and δ-tocopherol (Figure 3c–e) (*p*-value < 0.05).

**Figure 4:**
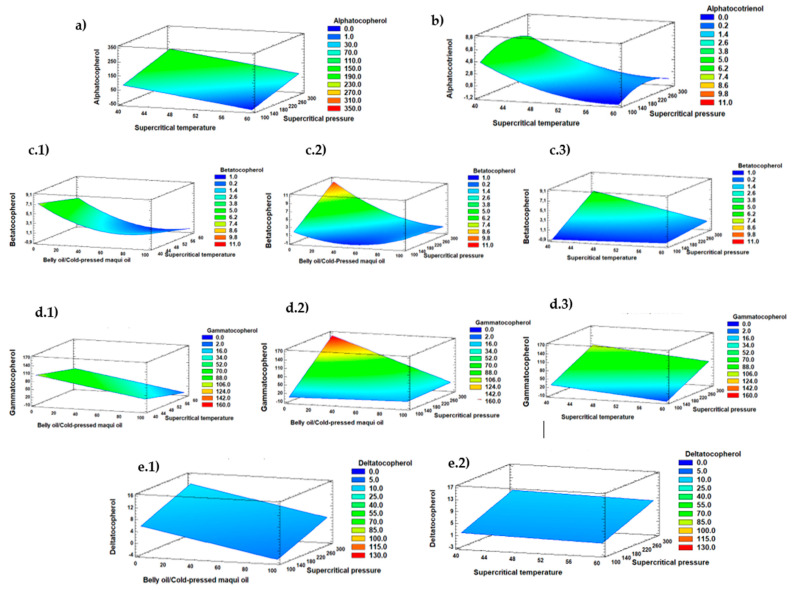
Estimated response surface diagrams for the response variable tocopherols: (**a**) α-tocopherol, (**b**) α-tocotrienol, (**c**) β-tocopherol, (**d**) γ-tocopherol, and (**e**) δ-tocopherol. Diagrams (**a**,**b**,**c.3**,**d.3**,**e.2**) show the effect of supercritical CO_2_ temperature (°C) vs. supercritical CO_2_ pressure (bar) at constant belly oil from rainbow trout/cold-pressed maqui seed oil (*w*/*w*) ratio. Diagrams (**c.1,d.1**) show the effect of belly oil from rainbow trout/cold-pressed maqui seed oil (*w*/*w*) ratio vs. supercritical CO_2_ temperature (°C) at supercritical CO_2_ pressure (bar). Diagrams (**c.2**,**d.2,e.1**) show the effect of belly oil from rainbow trout/cold-pressed maqui seed oil (*w*/*w*) ratio vs. supercritical CO_2_ pressure (bar) at constant supercritical CO_2_ temperature (°C).

Figure 4 shows the response surfaces of the effect of the independent variables, the belly oil from rainbow trout/cold-pressed maqui seed oil (*w*/*w*) ratio and supercritical CO_2_ pressure and temperature on α-tocopherol, α-tocotrienol, β-tocopherol, γ-tocopherol, and δ-tocopherol values. An increasing supercritical CO_2_ pressure and decreasing supercritical CO_2_ temperature resulted in higher content of α-tocopherol (a), α-tocotrienol (b), β-tocopherol (c), and γ-tocopherol (d). Additionally, β-tocopherol (c.2), γ-tocopherol (d.2), and δ-tocopherol (e.1) values showed an optimized value when the belly oil from rainbow trout/cold-pressed maqui seed oil ratio decreased, and the supercritical CO_2_ pressure increased. Thus, contents of β-tocopherol and γ-tocopherol showed the same behavior when the supercritical CO_2_ pressure and the belly oil/cold-pressed maqui seed oil ratio decreased, thus, obtaining the maximum yield for both tocopherols.

#### 2.2.4. Optimization of Enzymatic Interesterification Process Variables for Belly Oil from Rainbow Trout/Cold-Pressed Maqui Seed Oil Using Supercritical CO_2_ to Obtain an AL

Table 4 (Part a) presents the optimization of the enzymatic interesterification process variables, including the belly oil from rainbow trout/cold-pressed maqui seed oil (*w*/*w*) ratio, as well as the supercritical CO_2_ temperature (°C) and supercritical CO_2_ pressure (bar). Taking into account the information obtained from response variables for yield (Table 1), EPA and DHA content (Table 2), and tocopherol values (Table 3), the enzymatic interesterification process was optimized through RSM.

The optimal variables of the enzymatic interesterification process for yield were a 90.0/10.0 (*w*/*w*) belly oil from rainbow trout/cold-pressed maqui seed oil ratio, a 40.0 °C supercritical CO_2_ temperature, and a 299.69 bar supercritical CO_2_ pressure. The optimal value achieved for yield was 78.20% (Table 4, Part a). The high yield achieved under optimized conditions suggested that further exploration of the pressure variable, particularly at or near the optimal level identified (299.69 bar), could yield even better results than those seen in Table 1. These results indicated that the variables significantly impacted the yield, and careful adjustment of both pressure and temperature was crucial for maximizing enzymatic interesterification performance.

The optimal conditions for achieving the highest EPA and DHA contents were identified as a belly oil from rainbow trout/cold-pressed maqui seed oil ratio of 90:10 (*w*/*w*), supercritical CO_2_ temperature of 60.0 °C, and pressure of 299.69 bar, yielding EPA and DHA values of 6.43 and 6.25 g·100 g^−1^ total FAs, respectively. Results are in agreement with both the optimization (Table 4 Part a) and Table 2. The optimized conditions of process variables and the maximum concentration obtained for α-tocopherol were an oil ratio of 90/10 belly oil from rainbow trout/cold-pressed maqui seed oil, a supercritical CO_2_ temperature of 40.1 °C, a supercritical CO_2_ pressure of 300.00 bar; such values led to a maximum yield of 221.20 mg·kg^−1^ oil (Table 4, Part a).

The optimized value for α-tocotrienol was 6.43 g·100 g^−1^ total FAs. Although the maximum yield of α-tocotrienol is relatively low compared to α-tocopherol, the conditions set are still considered effective for extracting α-tocotrienol, which has its own health benefits.

The γ-tocopherol was optimized under specific conditions: an oil ratio of 10.8% belly oil from rainbow trout to 80.2% maqui seed oil, a temperature of 40.0 °C, and a pressure of 300.00 bar. Under these parameters, the maximum yield achieved was 166.18 mg·kg^−1^ of oil. This relatively high yield indicated that the optimized conditions effectively extracted γ-tocopherol, demonstrating a clear preference for a higher proportion of maqui seed oil.

In contrast, the maximum yield for δ-tocopherol was best achieved with slightly different conditions. The optimal oil ratio was 10/90 belly oil from rainbow trout to maqui seed oil, with the same temperature of 40.0 °C, but a slightly lower pressure of 299.72 bar. The maximum yield for δ-tocopherol was 12.69 mg·kg^−1^ oil. Similarly to the findings for β-tocopherol, these conditions suggested that the elevated concentration of δ-tocopherol was closely linked to the maqui seed oil, and the optimized pressure further enhanced its performance.

#### 2.2.5. Multiple Response Optimization of Enzymatic Interesterification and Desirability

**Figure 5:**
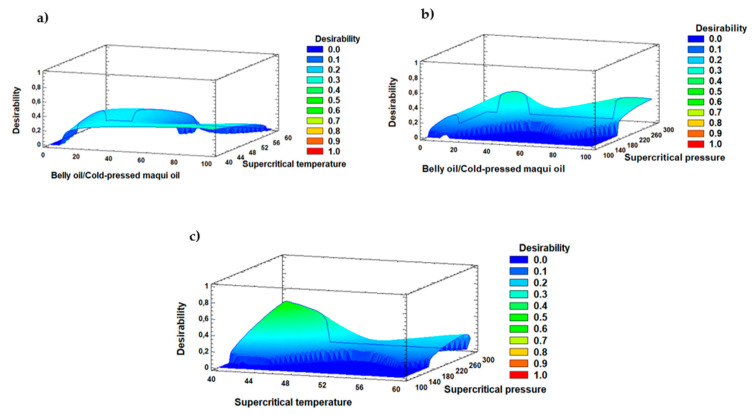
RSM graphs for the desirability value based on the belly oil from rainbow trout/cold-pressed maqui seed oil ratio, supercritical CO_2_ pressure, and supercritical CO_2_ temperature: (**a**) belly oil from rainbow trout/cold-pressed maqui seed oil vs. supercritical CO_2_ temperature, (**b**) belly oil from rainbow trout/cold-pressed maqui seed oil vs. supercritical CO_2_ pressure, and (**c**) supercritical CO_2_ temperature vs. supercritical CO_2_ pressure.

Table 4 (Part b) presents the results of the multiple response optimization, which assessed the combination of factor levels that simultaneously optimized all responses in the experimental design. The optimal combination of factor levels for maximizing the AL yield was determined with an 81.4/18.6 (*w*/*w*) ratio of belly oil from rainbow trout to cold-pressed maqui seed oil, a supercritical CO_2_ temperature of 40.0 °C, and a supercritical CO_2_ pressure of 299.99 bar. The optimized values for the responses variables yield, EPA content, DHA content, α-tocopherol, α-tocotrienol, β-tocopherol, γ-tocopherol, and δ-tocopherol were: 77.10%, 5.12 g·100 g^−1^ total FAs, 4.95 g·100 g^−1^ total FAs, 217.96 mg·kg^−1^ oil, 4.28 mg·kg^−1^ oil, 3.48 mg·kg^−1^ oil, 64.48 mg·kg^−1^ oil, and 6.39 mg·kg^−1^ oil, respectively. The desirability function value was 0.54, indicating that this combination of factors meets the desired criteria (Figure 5). A desirability value 0 represents an undesirable response, while a value of 1 indicates a highly desirable response [19].

#### 2.2.6. Experimental Validation of the Optimal AL Formulation

Table 4 (Part c) provides the experimental validation of the multiple response optimization for the response variables of Part b. The AL was validated using the optimized independent variables, i.e., 81.4/18.6 (*w*/*w*) (belly oil/cold-pressed maqui seed oil ratio), 40.0 °C (supercritical CO_2_ temperature), and 299.99 bar (supercritical CO_2_ pressure). The results of the corresponding response variables were as follows: yield of 78.8%, EPA and DHA content (g·100 g^−1^ total FAs) of 4.59 and 4.03, respectively, and tocopherol values (mg·kg^−1^ oil) of 101.76 (α-tocopherol), 0 (α-tocotrienol), 5.25 (β-tocopherol), 47.84 (γ-tocopherol), and 7.11 (δ-tocopherol).

Supplementary Table (Table S1) shows the linear (Y_2_, Y_3_, Y_4_, Y_6_, and Y_8_) and quadratic (Y_1_, Y_5_, and Y_7_) polynomial equations adjusted for the predicted models of oil yield (Y_1_) (Figure 1b), EPA content (Y_2_) (Figure 2b), DHA content (Y_3_) (Figure 2d), and the contents of α-tocopherol (Y_4_) (Figure 4a), α-tocotrienol (Y_5_) (Figure 4b), β-tocopherol (Y_6_) (Figure 4c), γ-tocopherol (Y_7_) (Figure 4d), and δ-tocopherol (Y_8_) (Figure 4e). Table S1 shows the regression coefficients of the first-order (Y_2_, Y_3_, Y_4_, Y_6_, and Y_8_) and second-order (Y_1_, Y_5_, and Y_7_) polynomial models for the different response variables. The results of fitting a multiple regression model describe the effect of the different process variables on the response variables considered for oil yield (%), EPA and DHA content (g·100 g^−1^ total FAs), and tocopherol concentration (mg·kg^−1^ oil) obtained from the interesterification of belly oil from rainbow trout and cold-pressed maqui seed oil under supercritical CO_2_.

### 2.3. Characterization of Physicochemical Properties of Cold-Pressed Maqui Seed Oil, Belly Oil from Rainbow Trout, and Antioxidant Lipid

#### 2.3.1. Measurement of Color Parameters (L*, a*, b*)

The results obtained from the measurement of color L*, a*, b* parameters are shown in Table 5. Significant differences (*p* < 0.05) among cold-pressed maqui seed oil, belly oil from rainbow trout, and the AL were observed for L*, a*, and b* values.

The color measurements of L*, a*, and b* are detailed in Table 5. Differences (*p* < 0.05) were observed among cold-pressed maqui seed oil, belly oil from rainbow trout, and AL for these parameters. For cold-pressed maqui seed oil, the L* value was 92.1 ± 0.0, placing it at the white end of the lightness scale; the a* value was −12.5 ± 0.0, reflecting a greenish hue; and the b* value was 51.8 ± 0.0, corresponding to a red color grade. Belly oil from rainbow trout presented an L* value of 75.3 ± 0.0, indicating a lighter shade of white, though less so compared to cold-pressed maqui seed oil. Its a* value was 32.3 ± 0.0, aligning closer to the red/purple end of the spectrum, and the b* value was 93.2 ± 0.0, suggesting a yellowish hue nearing orange. For the AL, the L* value was 78.4 ± 0.0, reflecting a white scale luminosity similar to that of belly oil from rainbow trout. The a* value was 14.2 ± 0.0, showing a slight inclination towards the red/purple spectrum, though to a lesser extent than belly oil. The b* value was 48.3 ± 0.0, placing it in the yellow range, close to orange, similar to cold-pressed maqui seed oil.

#### 2.3.2. Differential Scanning Calorimetry (DSC)

**Figure 6:**
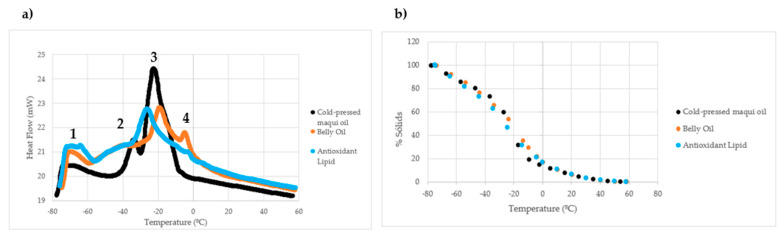
Differential scanning calorimetry. (**a**) Comparison thermogram of cold-pressed maqui seed oil, belly oil from rainbow trout, and antioxidant lipid. (**b**) Comparison of variation in solid content (%) as a function of temperature (°C) for cold-pressed maqui seed oil, belly oil from rainbow trout, and antioxidant lipid (LA). (1) peak with low-melting point TAG (LMTAG); (2, 3, 4) peaks with medium-melting point (MMTAG).

The thermogram of cold-pressed maqui seed oil, belly oil from rainbow trout, and the AL is shown in Figure 6a. For the cold-pressed maqui seed oil thermogram (black line), one peak with low-melting point TAG (LMTAG) was obtained, which is represented by number 1, and two peaks with medium-melting point (MMTAG) were detected, which are represented by numbers 2 and 3.

The belly oil from rainbow trout thermogram (orange line) shows one peak with LMTAG, represented by number 1, suggesting that it would correspond to n-3 LCPUFA (EPA and DHA) and three with MMTAG, represented by numbers 2, 3, and 4. Values corresponding to each peak of the oils are detailed in Table 6. Peaks 2 and 3 showed significant differences (*p* < 0.05) when comparing the two oils.

The thermogram of the AL (light blue line) shows two peaks with LMTAG that are represented by number 1. Three peaks with MMTAG are represented by numbers 2, 3, and 4.

Figure 6b illustrates the percentage of solid fat content melting over time at different temperatures. The oils exhibited similar behavior, and it can be observed that they are completely melted after temperatures of 40 °C, approximately.

Table 6 presents a summary of the DSC results for cold-pressed maqui seed oil, belly oil from rainbow trout, and AL. The table shows significant differences among the three oils for peaks 2 and 3, with the AL showing lower melting temperatures, i.e., −47 and −26 °C, respectively. Additionally, a significant difference was observed between the AL and the belly oil for peak 4.

#### 2.3.3. Chemical Properties

Table 7 shows the results of the quality analyses performed on cold-pressed maqui seed oil, belly oil, and AL. Significant differences (*p* < 0.05) were observed in free acidity, *p*-anisidine values, and total oxidation (TOTOX) values.

Peroxide values of 0.23 ± 0.06, 0.31 ± 0.03, and 0.71 ± 0.07 (mEq O_2_·kg^−1^ oil) were detected for cold-pressed maqui seed oil, belly oil from rainbow trout, and AL, respectively, indicating low primary oxidation development across these samples. A significant difference was observed in the FFAs content of the AL compared to cold-pressed maqui seed oil, suggesting minimal hydrolytic alteration of triacylglycerols in the AL. The *p*-anisidine values for cold-pressed maqui seed oil and belly oil from rainbow trout were 9.12 ± 0.00 and 6.86 ± 0.03, respectively, while the AL exhibited a significantly lower value of 0.16 ± 0.06. Conjugated dienes, which are indicative of primary oxidation formed through molecular rearrangement during hydroperoxide formation, were found to be very low in all oils. A high peroxide index typically would indicate the formation of conjugated dienes, which correspond to primary oxidation [58].

The AL showed a conjugated dienes value of 0.00, suggesting it is a fresh oil. Conjugated trienes, associated with secondary oxidation and related to *p*-anisidine values [58], were also negligible for both cold-pressed maqui seed oil and belly oil from rainbow trout, suggesting a low secondary oxidation development. The TOTOX value, which is calculated by combining both *p*-anisidine and peroxide value, provides an overall measurement of the total oxidation level of a sample [59].

#### 2.3.4. Analysis of the FA Profile

The results of the FA profile were obtained by Gas–Liquid Chromatography (GLC) are shown in Table 8. It is important to note that after the enzymatic interesterification process the Antioxidant Lipid incorporated part of the fatty acids from Cold-pressed maqui seed oil and Belly oil from rainbow trout, including the fatty acids EPA and DHA.

#### 2.3.5. Total Phenolic Content

The total phenolic content, measured using a gallic acid calibration curve, is presented in Table 9. Significant differences were detected among the three lipid samples (*p* < 0.05). Cold-pressed maqui seed oil exhibited a total phenolic content of 3.27 ± 0.15 mg gallic acid equivalents (GAEs)·g^−1^ oil, whereas belly oil had no detectable phenolic content, registering a value of 0 ± 0 mg GAE·g^−1^ oil. Thus, belly oil from rainbow trout analyzed in this study was found to be devoid of phenolic compounds; contrary, the AL depicted a valuable presence of such preservative compounds (i.e., 1.09 ± 0.06 mg GAE·g^−1^ oil).

#### 2.3.6. Determination of Tocopherol Compounds

The results for tocopherols in cold-pressed maqui seed oil, belly oil from rainbow trout, and the AL are presented in Table 10.

The tocopherol content results for cold-pressed maqui seed oil, belly oil from rainbow trout, and the AL are shown in Table 10. In cold-pressed maqui seed oil, the major components were α-tocopherol and γ-tocopherol, with values of 339.09 ± 5.15 and 135.52 ± 38.03 mg·kg^−1^ oil, respectively. Additionally, small amounts of β- and δ-tocopherol and traces of tocotrienol were detected. Both α-tocopherol and γ-tocopherol were the predominant compounds in the oils. These tocopherols exhibit anti-inflammatory activity, with γ-tocopherol being considered the most potent compound for scavenging free radicals (i.e., antioxidant capacity) [29].

In the AL, the major components were α-tocopherol and γ-tocopherol, with values of 101.76 ± 0.05 and 47.84 ± 0.22 mg·kg^−1^ oil, respectively. The α-tocotrienol value obtained was 0.00 ± 0.05 mg·kg^−1^ oil.

The AL values for both α-tocopherol and α-tocotrienol are lower than those predicted by the joint optimization, which were 216.76 and 4.23 mg·kg^−1^ oil, respectively. For β-tocopherol, a value of 5.25 ± 0.09 mg·kg^−1^ oil was obtained, which is higher than that found in belly oil from rainbow trout (0.20 mg·kg^−1^ oil). This result can be attributed to the presence of cold-pressed maqui seed oil in the AL. The β-tocopherol value in the AL is higher than the joint optimization prediction of 3.40 mg·kg^−1^ oil. For γ-tocopherol, the AL value was higher than that of belly oil from rainbow trout (18.01 mg·kg^−1^ oil). This is also explained by the presence of cold-pressed maqui seed oil in the AL. However, the AL value for γ-tocopherol is lower than the joint optimization prediction of 63.28 mg·kg^−1^ oil (Table 4, Part b). Meanwhile, the AL revealed a value of 7.11 ± 1.51 mg·kg^−1^ oil for δ-tocopherol, which is higher than both the joint optimization prediction of 6.39 mg·kg^−1^ oil (Table 4, Part b) and the values found for cold-pressed maqui seed oil and belly oil from rainbow trout separately. This can be explained on the basis of the results obtained in the 15 experiments shown in Table 3, where some values were also higher than those of cold-pressed maqui seed oil and belly oil from rainbow trout.

## 3. Discussion

The study employed RSM with a Box–Behnken design to investigate the enzymatic interesterification of cold-pressed maqui (*Aristotelia chilensis* (Mol.) Stuntz) seed oil and belly oil from rainbow trout (*Oncorhynchus mykiss*) from rainbow trout under supercritical CO_2_ conditions. The optimization of the enzymatic interesterification process aimed to maximize yield, EPA, DHA, and tocopherol contents.

The effect of the independent variables of the enzymatic interesterification process on yield indicated that a low supercritical CO_2_ temperature and high supercritical CO_2_ pressure were critical in order to achieve optimal levels of yield (*p* < 0.05). The results obtained in this study are attributed to the effect of supercritical CO_2_ pressure, as higher supercritical CO_2_ pressure (300 bar) resulted in the best yields, whereas lower supercritical CO_2_ pressure (100 bar) led to very low yields, except for experiment No. 10, which showed a 43.0% yield at 60 °C. The significant impact of supercritical CO_2_ pressure on yield aligns with previous studies that have explored similar enzymatic processes. Previous research [60,61] reported that the stability and the activity of enzymes exposed to carbon dioxide under high pressure depend on enzyme species, water content in the solution, and the pressure and temperature of the reaction system. Therefore, the higher pressures in supercritical CO_2_ systems enhanced solvation and improved the interaction between the enzyme and substrates, resulting in increased yields in the present study. This observation was reflected in the results, where the highest pressure of 300 bar consistently led to better yields compared to the lowest pressure of 100 bar, which resulted in negligible interesterification on the system.

The results presented in Table 1 demonstrated the significant influence of the belly oil from rainbow trout/cold-pressed maqui seed oil ratio (*w*/*w*) on the yield (%) of the enzymatic interesterification process. The experiments revealed that different oil ratios led to markedly distinct outcomes, attributed to the chemical properties of the oils and the specific operational conditions of the independent variables employed. The ratio of belly oil from rainbow trout to cold-pressed maqui seed oil affected the availability of substrates for the enzyme. However, according to the Pareto diagram, the ratio of belly oil from rainbow trout to cold-pressed maqui seed oil was not significant on the yield (*p* > 0.05). Supercritical CO_2_ pressure significantly affected the solubility and diffusivity of the reactants involved in enzymatic interesterification regarding yield (*p* < 0.05). At higher supercritical CO_2_ pressure (e.g., 300 bar), CO_2_ behaved more like a liquid, which increased the solvent power and enhanced the interaction between substrates and the enzyme. This resulted in greater yields, as evidenced by the higher yields observed in experiments No. 11, 12, and 8. Conversely, lower supercritical CO_2_ pressure (e.g., 100.0 bar) resulted in very low yields (1% to 3%), indicating that the supercritical CO_2_ pressure was not sufficiently effective in solubilizing the oils and facilitating the enzymatic reaction. While increasing supercritical CO_2_ temperature could enhance the reaction rate, it also had a negative effect on yield when combined with high supercritical CO_2_ pressure, likely due to enzyme denaturation or unfavorable interactions that disrupted the reaction balance. The results suggested that a lower supercritical CO_2_ temperature combined with a higher supercritical CO_2_ pressure optimized the yield, as shown in the Pareto diagram, where the temperature and pressure interaction had a reverse impact (*p* < 0.05) (Figure 1a). This effect was also observed in the estimated response surface, where a lower supercritical CO_2_ temperature and a high supercritical CO_2_ pressure led to a higher yield. Contrary, a high supercritical CO_2_ temperature and a low supercritical CO_2_ pressure led to lower performances (Figure 1b).

### 3.1. The Effect of the Independent Variables of the Enzymatic Interesterification Process on EPA and DHA Content

Supercritical CO_2_ pressure significantly affected the solubility and diffusivity of the reactants involved in the enzymatic interesterification regarding EPA and DHA content. At higher supercritical CO_2_ pressures, such as 300.0 bar, CO_2_ behaved more like a liquid, enhancing its solvent power and facilitating better interactions between the substrates and the enzyme. This resulted in higher yields of EPA and DHA content, as evidenced by the findings in experiments No. 8, 4, and 2 (Table 2). In contrast, lower supercritical CO_2_ pressures, like 100.0 bar, limited the solubilization of oils, leading to negligible amounts of EPA and DHA in experiments No. 5, 6, and 9 coinciding with the low performances observed in Table 1. The results can be explained by specific conditions of the interesterification process, such as the supercritical CO_2_ pressure, which may not have been optimal for facilitating the reaction. For example, if the supercritical CO_2_ pressure was too low, the solubility of the reactants could have been compromised, limiting the enzyme interaction with the substrates. The optimal response variable of EPA and DHA content of the enzymatic interesterification process were significantly influenced, both positively, with the highest belly oil from rainbow trout/cold-pressed maqui seed oil ratio (*w*/*w*) and the highest supercritical CO_2_ pressure, respectively. Pando et al. [62] reported that the optimized response for EPA and DHA contents required an increase in the n-3 LCPUFA/caprylic acid (CA) concentration ratio and supercritical CO_2_ time when they studied the triacylglycerol synthesis including EPA and DHA from rainbow trout (*O. mykiss*) belly flap oil and CA catalyzed by *Thermomyces lanuginosus* lipase under supercritical CO_2_. Dovale-Rosabal et al. [63] reported that the optimized response for EPA and DHA contents required an increase in supercritical CO_2_ temperature and pressure when they studied the effect of structured phenolic lipids with EPA/DHA and gallic acid against metabolic-associated fatty liver disease (MAFLD) in mice.

### 3.2. The Effect of the Independent Variables of the Enzymatic Interesterification Process on Tocopherol

Increased pressure values improved the solubility and diffusivity of the tocopherol compounds in the supercritical CO_2_ phase, leading to higher yields. The results indicated that tocopherol content was maximized at supercritical CO_2_ pressure around 200.0–300.0 bar. Conversely, elevated supercritical CO_2_ temperature generally reduced the tocopherol yields.

Higher supercritical CO_2_ temperature could degrade sensitive compounds, leading to lower antioxidant properties and compromised quality. In the present experiment, lower supercritical CO_2_ temperatures were associated with higher tocopherol levels, indicating a delicate balance between process efficiency and compound stability.

The proportion of belly oil from rainbow trout to cold-pressed maqui seed oil also significantly influenced tocopherol yields and played an important role in determining the content of tocopherols and the antioxidant properties. In agreement with the results obtained, a balanced proportion allowed the optimal extraction of tocopherols. Thus, the highest concentration of α-tocopherol (256.09 mg·kg^−1^ of oil) was achieved with a 50/50 ratio of belly oil from rainbow trout to maqui seed oil, indicating that this specific balance maximized the benefits of both types of oil. As the proportion of belly oil from rainbow trout increased, certain tocopherol concentrations were optimized, highlighting the importance of oil composition in the enzymatic interesterification process (Figure 4).

Additionally, the present study revealed that the optimized response of tocopherol compounds was obtained when increasing the supercritical CO_2_ pressure and decreasing the supercritical CO_2_ temperature (i.e., higher content of α-tocopherol, α-tocotrienol, β-tocopherol, and γ-tocopherol). Both α-tocopherol and γ-tocopherol were the predominant compounds in the oils. The optimization results are according to the findings of Sánchez et al. [64], who studied the optimization of oil and tocopherol extraction from (*Aristotelia chilensis* (Mol.) Stuntz) by supercritical CO_2_ procedure. These authors [64] reported that α-tocopherol was the predominant compound and an increase in supercritical pressure favored an increase in its concentration. This behavior was explained on the basis that, at pressures above 200 bar, the CO_2_ density generates high densities, and as the temperature increases, its solvent capacity decreases [65]. According to Yan et al. [53] the solubility and diffusivity of supercritical CO_2_ are related to its density and viscosity, and the density and viscosity can be adjusted by temperature and pressure. Consequently, the solubility and diffusivity of supercritical CO_2_ can be adjusted by controlling its temperature and pressure, so that the reaction can be adapted to different production requirements.

### 3.3. Multiple Response Optimization in the Current Study, the Optimized Values for Yield, EPA, DHA, α-Tocopherol, α-Tocotrienol, β-Tocopherol, γ-Tocopherol and δ-Tocopherol Contents

The method of multiple response optimization of the response variables by RSM is a faster and more economical method for gathering research results than classical one-variable-at-a-time or full-factorial experimentation. This method has been successfully used in many optimization studies [62,64,66,67,68,69] and is the preferred experimental design for fitting polynomial models to analyze the response surfaces of multi-factor combinations. The RSM-based optimization identified that maximizing yield and EPA, DHA, and tocopherol values required higher belly oil from rainbow trout ratios and supercritical CO_2_ pressures for LA. The optimal combination of factor levels for maximizing all responses in the experimental design and the optimized values for the different response variables, i.e., yield, EPA, DHA, α-tocopherol, α-tocotrienol, β-tocopherol, γ-tocopherol, and δ-tocopherol values to obtain the AL is observed in Table 4, Part b. Supplementary Table (Table S1) shows the results of ANOVA and fitting and the multiple regression model describing the effect of the different process variables on the response variables considered for oil yield (%), EPA and DHA content (FA g·100 g^−1^ total FAs), and tocopherol concentration (mg·kg^−1^ oil) obtained from the interesterification of belly oil from rainbow trout and cold-pressed maqui seed oil under supercritical CO_2_.

The experimental validation of the multiple response optimization for the response variables of the antioxidant lipid was validated using the optimized independent variables as the following was observed Table 4 Part c.

When comparing and analyzing the results from Table 3 regarding the optimization of the enzymatic interesterification process for belly oil from rainbow trout and cold-pressed maqui seed oil (Table 4, Part a), a remarkable increase in α-tocotrienol in Table 5 compared to Table 3 is observed, indicating that the optimized process significantly enhances α-tocotrienol extraction. The switch in oil ratio to favor maqui seed oil significantly impacts the extraction of β-tocopherol, indicating its higher concentration in this oil compared to belly oil from rainbow trout. The optimized pressure also aids in its performance.

Enzymatic interesterification modified the triacylglycerol (TAG) composition of oils, promoting the production of lipids with antioxidant properties. The supercritical CO_2_ acted as an efficient solvent, enhancing the solubilization of TAG from belly oil from rainbow trout and the bioactive compounds from maqui seed oil. This improved solubility facilitated a more effective contact between the substrates and the enzyme. In the supercritical environment, the enzyme is bound to the TAGs. The controlled high pressure and temperature maintained the enzymatic activity, which aided in the catalytic process. The enzyme catalyzed the cleavage of ester bonds in the TAGs, producing free fatty acids and glycerol and subsequently esterification occurred where the FFAs generated were then able to reattach to glycerol or to other TAGs, resulting in new combinations of FAs. This mechanism allowed the omega-3 FAs (EPA and DHA) from the belly oil from rainbow trout to integrate into the lipid structures of the maqui seed oil. The outcome of this process was the formation of new TAGs that combined the nutritional and antioxidant properties of both oils. These modified lipids exhibited improved oxidative stability due to the presence of tocopherols and other antioxidants derived from maqui seed oil.

By varying the ratios of the oils and adjusting the operational conditions (pressure and temperature), it was possible to optimize the proportions of FAs and bioactive compounds in the final product, as the AL, thereby maximizing its antioxidant capacity.

The behavior exhibited by the enzyme *Thermomyces lanuginosus* during interesterification aligns with the observations of authors 1–3, who indicate that water-insoluble substrates can be transformed by enzymes in non-aqueous media [1,2,5]. Enzymes such as proteases, lipases, peroxidases, and esterases are stable and active in organic solvents, which has significantly broadened their applications as highly enantioselective catalysts in organic synthesis [70,71,72].

The process resulted in several significant advantages. On the one hand, the incorporation of a natural antioxidant and the combination of omega-3 FAs enhanced the product resistance to oxidation, as well as the synergistic effect of EPA and DHA with antioxidants increased the beneficial properties of the resulting oil, making it suitable for functional food applications and dietary supplements.

### 3.4. Comparative Physico-Chemical Analysis of Oils

For the color measurements of L*, a*, and b* parameters, significant differences (*p* < 0.05) were observed among cold-pressed maqui seed oil, belly oil from rainbow trout, and the AL. For the AL, the L* value was 78.4 ± 0.0, reflecting a white scale luminosity similar to that of belly oil from rainbow trout. The a* value was 14.2 ± 0.0, showing a slight inclination towards the red/purple spectrum, though to a lesser extent than belly oil from rainbow trout. The b* value was 48.3 ± 0.0, placing it in the yellow range, close to orange, similar to cold-pressed maqui seed oil. The results of color measurements showed that cold-pressed maqui seed oil was different from those reported by Bastías-Montes et al. [29] and this also happened for belly oil results compared to the results of Pando et al. [62]. This could be due to the difference in the origin of oils.

For the cold-pressed maqui seed oil thermogram, one peak with low-melting-point TAG (LMTAG) was obtained, and two peaks with medium-melting-point (MMTAG). The belly oil thermogram showed one peak with LMTAG, suggesting that it would correspond to n-3 LCPUFA (EPA and DHA) and three with MMTAG. The thermogram of the AL shows two peaks with LMTAG suggesting that it would correspond to n-3 LCPUFA (EPA and DHA) similarly to the thermogram obtained in belly oil from rainbow trout and three peaks with MMTAG. Siddique et al. [73] studied different oils and oil blends including palm olein, canola oil, marigold oil, and soybean oil. The thermogram of cold-pressed maqui seed oil resembled maravilla oil and the peaks are similar to those obtained by Siddique et al. [73] since they appear at similar temperatures. This can be explained on the basis that in cold-pressed maqui seed oil and sunflower oil the FA content is similar, i.e., with a high content of C18:2n-2 (linoleic acid) and C18:1n-9 (oleic acid) [74].

In fish oil, the peaks with low-melting-point TAGs (LMTAGs) found at a low temperature melting point are characteristic of TAGs with FAs of low-melting-point and, therefore, these peaks can be attributed to the presence of EPA and DHA. It can be observed that the thermogram of obtained AL is quite similar to that of belly oil from rainbow trout, as it has the same melting peaks found at low temperatures of the thermogram, which are characteristic of n-3 LCPUFA, attributable to the presence of EPA and DHA [75,76]. A similar result for this thermogram was obtained by Sathivel [77] from unrefined red salmon oil. Peak 4 of the AL was not as pronounced as in belly oil. This could be due to the AL being a mixture of belly oil from rainbow trout and cold-pressed maqui seed oil, where peak 4 is not observed in the latter.

In terms of quality analyses, the analysis of free acidity indicates the modification of TAGs, which may occur through enzymatic action or chemical hydrolysis [78]. According to RSA specifications, where the maximum is 0.25% free acidity, expressed as oleic acid, the present results of the oils comply with such requirements [79]. A significant difference was observed in the FFAs content of the AL compared to cold-pressed maqui seed oil, suggesting minimal hydrolytic alteration of TAGs that exhibited a lower value (i.e., 0.16 ± 0.06). Peroxide values of 0.23 ± 0.06, 0.31 ± 0.03, and 0.71 ± 0.07 (mEq O_2_·kg^−1^ oil) were measured for cold-pressed maqui seed oil, belly oil from rainbow trout, and AL, respectively, indicating a low primary oxidation development in these samples. The cold-pressed maqui seed oil, belly oil from rainbow trout, and the AL comply with the RSA requirements. It is found that the RSA specifies: “At the time of production, the maximum peroxide limit shall be 2.5 meq of peroxide oxygen·kg^−1^ fat, and 10 meq of peroxide oxygen·kg^−1^ fat during its shelf life, when stored as indicated on the labeling” [79]. The *p*-anisidine value of cold-pressed maqui seed oil obtained a value similar to that detected by Raza et al. [80] in a refined sunflower oil in the sixth week of autoxidation. It should be noted that the cold-pressed maqui seed oil in this work was not refined. Currently, in the Chilean RSA, there is no limiting value for *p*-anisidine, but Matthäus [81] indicated in his work that in the industry, in general, a limit for *p*-anisidine of 10 is accepted. Therefore, cold-pressed maqui oil would meet that value. In contrast, for fish oils there is a Codex Alimentarius standard (CXS 329-2017) in which the *p*-anisidine index has to be ≤20 [82]; therefore, the current belly oil from rainbow trout meets this standard. The *p*-anisidine value for the AL exhibited a significantly lower value of 0.16 ± 0.06 in comparison with belly oil from rainbow trout and cold-pressed maqui seed oil. Conjugated dienes were found to be very low in all oils, reflecting a minimal primary oxidation development. The AL had a conjugated diene value of 0.00 ± 0.00, suggesting that it is a fresh oil. Conjugated trienes, associated with secondary oxidation and related to *p*-anisidine values [74], were also negligible for both cold-pressed maqui seed oil and belly oil from rainbow trout, suggesting a low secondary oxidation development. The AL also exhibited a conjugated triene value of 0.00 ± 0.00, similar to that of conjugated dienes. The TOTOX value for the AL was 1.57 ± 0.20, which reflects a significant difference when compared to the values of cold-pressed maqui seed oil and belly oil from rainbow trout. This TOTOX value is consistent with the low peroxide value and the low conjugated diene and triene values, suggesting a minimal oxidation development in the AL. In addition, the value obtained for the AL complies with the Codex Alimentarius, which indicates that the TOTOX value for a fish oil must be ≤26 [82].

The FA profiles of cold-pressed maqui seed oil, belly oil from rainbow trout, and AL were analyzed using GLC, thus, revealing distinct differences in their compositions. In this study, the results suggest that cold-pressed maqui seed oil is rich in monounsaturated and polyunsaturated FAs, contributing to its potential health benefits and stability as a nutritional oil. Bastías-Montes et al. [29], who analyzed a cold-pressed maqui seed oil, obtained similar results, where linoleic acid was predominant. Marmesat et al. [83] found in a high-oleic sunflower oil that oleic acid was the most abundant FA. Thus, considering that this oil has an especially high oleic content, the presence of this FA was higher than in the present study. Regarding the belly oil, Dovale-Rosabal et al. [84] obtained a similar profile, but with some quantitative differences; thus, lower EPA and DHA levels were obtained for a refined commercial salmon oil when compared to the belly oil of the current study. For belly oil and the AL, the EPA and DHA values can be considered within the ranges established by the Codex [82] for farmed salmon oil. The presence of these long-chain n-3 FAs in belly oil suggests its potential cardiovascular benefits and enhanced nutritional value. The AL, which was derived from an enzymatic interesterification process involving a mixture of belly oil from rainbow trout and cold-pressed maqui seed oil, displayed a total of 24 FAs. Overall, these results highlight the impact of different oil sources and processing methods on the FA profile. Cold-pressed maqui seed oil is distinguished by its high content of oleic and linoleic acids, whereas belly oil offers a more varied FA profile with substantial n-3 FAs. The AL serves as a compromise between these two sources, reflecting the influence of the interesterification process on the FA composition. Future studies could explore the implications of these FA profiles on health and stability, particularly in relation to the potential applications of the current AL.

For phenolic compounds, a similar result than in the present one was reported in the study by Rahim al. [85], in which they analyzed fish oil from rohu (*Labeo rohita*) (rohu is a freshwater fish and belongs to the carp family) [86] that was not refined, resulting in a value of 0.03 mg GAE·g^−1^ of oil. Regarding the cold-pressed maqui seed oil, similar results were found in the study by Parry et al. [87] for cold-pressed berry oils, with blueberry oil having the highest value at 1.00 mg GAE·g^−1^ oil.

The AL contained α-tocopherol (101.76 ± 0.05 mg·kg^−1^) and γ-tocopherol (47.84 ± 0.22 mg·kg^−1^) but did not detect α-tocotrienol. The β-tocopherol concentration (5.25 ± 0.09 mg·kg^−1^) was higher than in belly oil, probably due to the inclusion of cold-pressed maqui oil. The δ-tocopherol concentration (7.11 ± 1.51 mg·kg^−1^) was notably higher than in both cold-pressed maqui seed oil and belly oil from rainbow trout, as well as above the joint optimization prediction. This increase reflects the impact of integrating different oil sources in the formulation of the AL.

Regarding the results of tocopherols from cold-pressed maqui seed oil, Bastías-Montes et al. [29] reported lower values than in the current study for α- and γ-tocopherols, with values of 169 mg·kg^−1^ oil and 57 mg·kg^−1^ oil, respectively. Such authors also reported high values for α-tocotrienol (324 mg·kg^−1^ oil) and δ-tocotrienol (54 mg·kg^−1^ oil), this latter not being detected in this study. Meanwhile, Sánchez et al. [64] reported, for freeze-dried maqui seed oil, values of 347 mg·kg^−1^ oil for α-tocopherol, 28 mg·kg^−1^ for α-tocotrienol, traces for β-tocopherol, 50 mg·kg^−1^ for γ-tocopherol, and traces for δ-tocopherol. When compared to fish oil, these values were similar to those found by Ortiz et al. [88] in Coho salmon (*O. kisutch*), which belongs to the same family and genus as the species studied in this study, rainbow trout (*O. mykiss*). In the case of α-tocopherol, the belly oil from rainbow trout had a value of 191.80 mg·kg^−1^ oil with the closest comparable value being in Diet I (Control), which had a value of 209.3 mg·kg^−1^ oil. Ortiz et al. [88] did not report results for α-tocotrienol, probably because this compound, when present in such small quantities, may not be detected by the equipment used. However, α-tocotrienol was found in belly oil, from rainbow trout although in a low concentration (2.02 mg·kg^−1^ oil). For β-tocopherol, a very low value was obtained in belly oil from rainbow trout (0.20 mg·kg^−1^ oil), with Ortiz et al. [88] detecting traces. This result is likely due to β-tocopherol being minimally deposited in fish muscles [89]. For γ-tocopherol, the belly oil from rainbow trout had a value of 18.01 mg·kg^−1^ oil, which was similar to that found in Diet III (tocopherols and rosemary extract) with a value of 19.6 mg·kg^−1^ oil. Finally, for δ-tocopherol, the belly oil from rainbow trout showed a value of 1.40 mg·kg^−1^ oil, which is similar to the value obtained for Diet III (tocopherols and rosemary extract), which was 1.1 mg·kg^−1^ oil. Ortiz et al. [88] explained that δ-tocopherol is deposited in lower amounts compared to α- and γ-tocopherol because, unlike these two tocopherol compounds, it is consumed more rapidly to protect lipids from lipid peroxidation.

It should be noted that the current rainbow trout (*O. mykiss*) was also fed with antioxidants in its diet; consequently, similar results to those by Ortiz et al. [88] were expected. The differences in values may be primarily due to the species studied. Compared to the study by Ortiz et al. [88], the α-tocopherol value in the AL is closer to that of Diet I (Control). The value obtained for α-tocotrienol was consistent, while the value for β-tocopherol was higher, as they found only trace amounts. For γ-tocopherol, the value was higher than that of Diet II (excess of tocopherols). Finally, for δ-tocopherol, the closest value was the one obtained for Diet II (excess of tocopherols), which was approximately 4 mg·kg^−1^ oil.

## 4. Materials and Methods

### 4.1. Raw Material and Reagents

The abdominal region, referred to as the belly from rainbow trout (*O. mykiss*), was obtained from the aquaculture company Salmones Antártica S.A. (Puerto Montt, Chile), where the fish were raised and processed at their plant in Chiloé, Chile. The processing steps at the plant were as follows: the rainbow trout were received at 13.5 °C and stored in bins with a liquid ice FLOW-ICE^®^ Pty Ltd. (Mandurah, Australia) solution at −1.8 °C within a cold chamber maintained at 0 °C. After 80 h, the fish were removed from this chamber at −1 °C for processing. Following this, they were mechanically filleted and trimmed according to the desired specifications, resulting as the belly by-product. After processing, the belly muscle showed a temperature of 3.4 °C. It was then packaged in 1 kg bags and vacuum sealed. For transport, the belly was frozen at −24 °C, stored with gel packs in a thermal box, and shipped to the Faculty of Chemical and Pharmaceutical Sciences of the University of Chile (Santiago, Chile) overnight. Upon arrival at 11:00 AM, 50 kg of the belly were received and stored at −80 °C in a deep freezer until use.

Cold-pressed maqui (*Aristotelia chilensis* (Mol.) Stuntz) seed oil was purchased from the company De Castañas y Amores (Santiago, Chile); it was preserved at −80 °C and protected from light. Lipozyme^®^ TL IM, a 1,3-specific lipase from the fungus *Thermomyces lanuginosus*, was used for enzymatic interesterification; for it, the enzyme was immobilized on a non-compressible silica gel carrier (Novozymes A/S (Copenhagen, Denmark), obtained from the company Merck S.A. (Santiago, Chile).

Indium Standard, Internal Standard Methyl Tricosanoate (CH_3_(CH_2_)_21_ COOCH_3_ NU-CHEK) and Reference GLC-463 for GLC were purchased from Nu-Chek-Prep, Inc. (Elysian, MN, USA). Liquid CO_2_ with a purity of 99.9% for enzymatic interesterification, as well as H_2_ and N_2_ for GLC, were obtained from GasLab-Linde (Santiago, Chile). Gallic acid, Folin–Ciocalteu reagent, AAPH (2,2′-azobis (2-methylpropionamidine) dihydrochloride), and Trolox (6-hydroxy-2,5,7,8-tetramethylchroman(E)-2-carboxylic acid) were purchased from Sigma Aldrich, Chemie GmbH (Steinheim, Germany), and other chemical reagents were purchased from CalbioChem Merck (Santiago, Chile). The α, β, γ, δ-tocopherol and α-tocotrienol standards were purchased from CalbioChem^®^ (Darmstadt, Germany). Fluorescein was purchased from Merck KGaA (Darmstadt, Germany).

### 4.2. Extraction of Belly Oil from Rainbow Trout

The extraction of oil from the belly of rainbow trout was performed using the modified Radin method [16,17,62]. One hundred grams of belly, previously thawed in the refrigerator overnight, were weighed before use and ground in a food processor. In a beaker, a solvent mixture of 1800 mL hexane and isopropanol (3:2, *v*/*v*) ratio was added to the ground tissue. It was then filtered under a vacuum using a Büchner funnel with Whatman No. 1 filter paper. The solid phase was discarded. The liquid phase from the vacuum filtration was extracted and mixed with 700 mL of 3% anhydrous Na_2_SO_4_ solution. The mixture was stirred with a magnetic stirrer for 10 min. After stirring, the mixture was filtered again under vacuum using a Büchner funnel with Whatman No. 1 filter paper, and the solid phase was discarded. The filtered solution was transferred to a separation funnel to separate the organic phase from the aqueous phase. The organic phase, which was of interest, was collected. To remove the solvent (hexane–isopropanol, 3:2 *v*/*v*) from the organic phase, a rotary evaporator was used at 40 °C for approximately 2 h or until the solvent was evaporated. After the solvent was removed, the crude oil was obtained. Nitrogen was then added to the crude oil, and it was stored in an ultra-freezer at −80 °C. The extraction yield was calculated according to the following Equation (1):

(1) $$(\%{)Y}_{B}=\left(\frac{P_{O}}{P_{B}}\right)\times 100$$

where %*Y_B_* is the percentage of extracted oil yield, *P_O_* is the weight of the extracted oil (g), and *P_B_* is the total weight of the belly used for each extraction (g).

### 4.3. Enzymatic Interesterification Under Supercritical CO_2_ of Belly Oil and Cold-Pressed Maqui Seed Oil

Enzymatic interesterification was conducted using *Thermomyces lanuginosus* lipase under supercritical *CO_2_* combining belly oil from rainbow trout and cold-pressed maqui seed oil to obtain an AL. For this process, a high-pressure reactor, specifically the Speed SFE system model 7071 supercritical CO_2_ equipment (Spe-edTM SFE Applied Separation, Allentown, PA, USA), was employed. A stainless-steel column with a capacity of 10 g of sample was used into which the reaction substrates (belly oil from rainbow trout and cold-pressed maqui seed oil) and the enzyme were introduced. The column was securely placed in the supercritical reactor chamber, and the temperature and pressure were adjusted to achieve the supercritical CO_2_ conditions according to the established experimental design (Table 1).

### 4.4. Experimental Design Using Response Surface Methodology (RSM) for Enzymatic Interesterification Under Supercritical CO_2_ Condition

For the experimental design, a Box–Behnken design was utilized using RSM according to Myers and Montgomery [90]. Three independent variables were studied: the belly oil from rainbow trout/cold-pressed maqui seed oil ratio (*w*/*w*) at 10/90, 50/50, and 90/10 values; supercritical CO_2_ temperature at 40, 50, and 60 °C; and supercritical CO_2_ pressure at 100, 200, and 300 bar. The reaction time was kept constant at 2 h for the enzymatic reaction and 2 h for extraction. The response variables measured included yield, concentrations of EPA, DHA, and tocopherols.

The experimental design followed a RSM with a Box–Behnken design, detailed in Table 1. A total of 15 experiments were conducted, including 3 central point experiments to estimate experimental error. These experiments were performed in random order to minimize variability in the observed responses.

The antioxidant-structured lipids obtained were stored at −80 °C in amber bottles with nitrogen to prevent oxidation. The response variables were yield, EPA, DHA, and tocopherol contents of the interesterified antioxidant lipid under supercritical *CO*_2_.

### 4.5. Optimization of Enzymatic Interesterification Process Variables of Belly Oil from Rainbow Trout/Cold-Pressed Maqui Seed Oil Using Supercritical CO_2_ to Obtain an Antioxidant Lipid

The independent variables of the enzymatic interesterification process (belly oil from rainbow trout/cold-pressed maqui seed oil (*w*/*w*), supercritical CO_2_ temperature (°C), supercritical CO_2_ pressure (bar)) were optimized (Table 5). Using the data obtained for the response variables of yield, EPA, DHA and tocopherol values, the enzymatic interesterification process was optimized by RSM according to Hill and Hunter [91], Myers and Montgomery [90], and Liu et al. [92]. The data allowed us to build predictive quadratic polynomial models in terms of their regression coefficients for the independent variables and to establish the combination of variables that provided a theoretical or predicted optimum with a maximum of the response variables. Multiple regression models were fitted to the response variables by discarding non-significant (*p* > 0.05) terms to obtain response surfaces. A quadratic polynomial regression model was assumed for predicting individual Y variables, expressed in the following Equation (2):

(2) $$Y=\beta_{0}+\sum_{i=1}^{k}\beta_{i}X_{i}+\sum_{i=1}^{k}\beta_{ii}X_{i}^{2}+\sum_{i=1}^{k}\sum_{j=1}^{k}\beta_{ij}X_{i}X_{j}+\varepsilon$$

In the fitted equation, *Y* is the response variable, $\beta_{0}$ is the intercept term in the regression model, $\beta_{i}$ is the coefficient for each main effect, $\beta_{ij}$ is the coefficient for interactions between variables *i* and *j*, $\beta_{ii}$ is the coefficient for quadratic terms that represent the curvature of the surface or non-linearity in the relationship between variables, X represents each independent variable, *ε* is the error term, accounting for variability not explained by the model, and *k* is the number of independent variables.

A multiple response optimization was conducted to evaluate the combination of experimental factor levels that simultaneously optimized all responses of the experimental design. This method is used to find the best combination of experimental factor levels that optimizes multiple response variables simultaneously.

The desirability function, which varies within the range of 0 to 1, was used as a tool to evaluate how well a combination of factors meets the desired criteria. A value of 0 indicates an undesirable response, while a value of 1 indicates a desirable response [62,63]. By combining all dependent variables, a theoretical value was obtained, and the predicted optimum for yield (%), EPA, DHA, and tocopherol contents (mg·kg^−1^ oil) was established. The statistical software STATGRAPHICS Centurion XVI Version 16.1.03 (32-bit) (Leesburg, VA, USA) was used.

### 4.6. Validation of the Optimal Antioxidant Lipid Formulation and Comparison with Cold-Pressed Maqui Seed Oil and Belly Oil from Rainbow Trout

After obtaining the optimal AL of interest through enzymatic interesterification with the optimal conditions of the belly oil from rainbow trout/cold-pressed maqui seed oil ratio (*w*/*w*), supercritical CO_2_ temperature (°C), and pressure (bar), validation was carried out. The AL formulation obtained from the optimum point of the experimental design was experimentally validated considering the predictive optimum conditions of the belly oil from rainbow trout/cold-pressed maqui seed oil ratio (*w*/*w*), supercritical pressure, and supercritical temperature. Thus, the optimal AL was validated and characterized by calculating performance with the equation shown in (3), determining yield, EPA and DHA content by CGL, and measuring tocopherols as described in Table 4. Part c.

### 4.7. Characterization of Physicochemical Properties of Cold-Pressed Maqui Seed Oil, Belly Oil from Rainbow Trout and Antioxidant Lipid

For each experiment, the yield obtained was calculated according to the following Equation (3):

(3) $$(\%{)R}_{I}=\left(\frac{P_{AI}}{P_{BI}}\right)\times 100$$

where R*_I_* is the percentage of interesterified AL yield (%), P*_BI_* is the weight of oil mixture before the interesterification process (g), and P*_AI_* is the weight of oil mixture after the interesterification process(g).

Measurement of color parameters (L*, a*, and b*) was evaluated in the cold-pressed maqui seed oil, belly oil from rainbow trout, and the AL using the AOCS method (Cc 13e-92) [59] with a Lovibond Colorimeter (Amesbury, UK) according to the Commission Internationale de l’Éclairage (CIE-International Commission on Illumination) standards. The L* value indicated luminosity, a*: red/green coordinates (+red, -green), and b*: yellow/blue coordinates (+yellow, -blue).

The thermal properties of cold-pressed maqui seed oil, belly oil from rainbow trout, and the AL were obtained by thermogram analysis with PerkinElmer DSC 6000 equipment (Waltham, MA, USA). The thermograms were analyzed with the Pyris Player Software computer program, version 11.0.0.0449, where the temperatures (°C) of the beginning of the melting curve (T_Onset_), the maximum of the peaks (T_Peak_), and the end of the melting curve (T_Endset_) were obtained. Furthermore, the enthalpy of fusion (ΔH) was obtained from T_Onset_ and T_Endset_ of the sample (J·g^−1^) [64,93].

Official AOCS procedures were employed for the cold-pressed maqui seed oil, belly oil from rainbow trout, and AL damage assessment [59]: Free fatty acid (Ca 5a-40), peroxide value (Cd 8b-90), *p*-anisidine value (Cd 18-90), TOTOX value (Cg 3-91), and conjugated diene and triene formation at 233 nm and 268 nm, respectively (Ti 1a-64).

The composition of FAs of cold-pressed maqui seed oil, belly oil from rainbow trout, and the AL was determined by converting them into FA methyl esters (FAMEs) through alkaline and acid methylation according to the method described by the IUPAC [94]. The analysis of the resulting FAMEs was conducted following AOCS Ce 1j-7 [59]. A GLC HP5890 series II (Palo Alto, CA, USA) equipped with a flame ionization detector, a split injection system, and a capillary column (100 m × 0.25 mm i.d. × 0.2 µm) SP^TM^-2560 (Supelco, Bellefonte, PA, USA), was used. The initial oven temperature was set at 160 °C and maintained for 3 min, then increased at a rate of 1 °C·min^−1^ until reaching 230 °C. Both the injector and flame ionization detector temperatures were set at 240 °C. Hydrogen was used as the carrier gas. The signal emitted by the detector was analyzed using Data ApexClarity^TM^ software M021/80S (DataApex Ltd., Prague, Czech Republic). For qualitative determination, the retention times of the samples were compared to those of a previously injected standard (GLC-463, Nu-Chek Prep, Elysian, MN, USA). The quantification of all individual FAs (g·100 g^−1^ total FAs) was achieved using methyl tricosanoate (C23:0 methyl ester), an internal standard according to the AOCS method [59].

The total phenolic content in cold-pressed maqui seed oil, belly oil from rainbow trout, and the AL was determined spectrophotometrically using the Folin–Ciocalteu method as described by Fuentes et al. [95], using an Unicam UV/Vis spectrophotometer model UV3 (Cambridge, UK). The calibration curve was constructed using six different concentrations of a standard solution of gallic acid (CalbioChem Merck, Santiago, Chile), ranging from 50 to 500 μg·mL^−1^ (R^2^ = 0.9979). The results were expressed as μg gallic acid equivalents (μg GAE·g^−1^ oil).

The presence of tocopherol compounds in cold-pressed maqui seed oil, belly oil from rainbow trout, and the AL was determined by high-performance liquid chromatography (HPLC) according to the AOCS standard method Ce 8-89 [59] using an HPLC consisting of a Merck-Hitachi pump L-6200A (Merck, Darmstadt, Germany), a Rheodyne 7725i injector with 20 μL sample loop, a LiChro-CART Superspher Si 60 column (25 cm × 4 mm id, 5 μm particle size; Merck, Darmstadt, Germany), a Hitachi Chromaster 5440 fluorescence detector, and a PC with Clarity chromatography software version 2.4.1.43 (Prague, Czech Republic), to processing the chromatographic signals detected at excitation and emission wavelengths of 290 nm and 330 nm, respectively. For the qualitative and quantitative analyses, commercial standards of tocopherol compounds (α-, β-, γ-, and δ-tocopherol and α-tocotrienol) obtained from CalbioChem^®^ (Darmstadt, Germany) were used. Results were expressed as mg tocopherols·kg^−1^ oil.

### 4.8. Statistical Analysis

ANOVA of the regression parameters and model fitting were performed (*p* < 0.05), and regression analysis was applied to the data using RSM. The estimated response surfaces were developed using the fitted quadratic polynomial equations obtained from the response surface regression analysis, with the independent variable that had the least effect on the response held constant while varying the levels of the other two variables [67,68]. A multiple-response optimization was conducted to assess the combination of experimental factors that simultaneously optimized several responses. As a result, the maximization of the desirability function, which ranges from 0 to 1, was achieved [68]. Analyses were performed in triplicate, and the standard deviation of each sample was considered. The software used for the analysis was STATGRAPHICS Centurion XVI Version 16.1.03 (32-bit) (Leesburg, VA, USA) as a tool for statistical analysis and optimization. The AL obtained was experimentally validated by applying the conditions proposed in the theoretical optimum.

## 5. Conclusions

According to the present study, it was possible to extract oil from rainbow trout belly, reaching a maximum yield of 50.4%. Values obtained as a result of the quality analysis including peroxide, free fatty acid, *p*-anisidine, conjugated diene and triene, and TOTOX values comply with the regulatory arranged values depending on the type of oil, i.e., RSA (both oils), Codex Alimentarius (fish oil), and IOC (vegetable oil). In the analysis of FAs by GLC, the presence of oleic (C18:1n-9) and linoleic (C18:2n-6) acids was highlighted in belly oil from rainbow trout and cold-pressed maqui seed oils. In addition, in belly oil, the EPA and DHA content was found to be highly valuable and did not contain phenol compounds. In contrast, a high value for total phenols was obtained for cold-pressed maqui seed oil compared to olive oil and cold-pressed oils from different berries. Regarding the determination of tocopherol compounds, the major ones were α-tocopherol and γ-tocopherol in belly oil from rainbow trout and cold-pressed maqui seed oil, thus, offering potential health benefits. From the enzymatic interesterification of belly oil with cold-pressed maqui seed oil under supercritical CO_2_, it can be concluded that the best performance was determined by employing an 81.4/18.6 (*w*/*w*) ratio of belly oil from rainbow trout and cold-pressed maqui seed oil, a supercritical CO_2_ temperature of 40.0 °C, and a supercritical CO_2_ pressure of 299.99 bar. The results of the corresponding response variables were as follows: yield of 78.8%, EPA and DHA contents of 4.59 and 4.03 g·100 g^−1^ TFAs, respectively, and values of 101.76 mg·kg^−1^ oil for α-tocopherol, 0 mg·kg^−1^ oil for α-tocotrienol, 5.25 mg·kg^−1^ oil for β-tocopherol, 47.84 mg·kg^−1^ oil for γ-tocopherol, and 7.11 mg·kg^−1^ oil for δ-tocopherol; such values suggest potential health benefits and effective preservation of the new AL.

Therefore, it was possible to synthesize a new lipid rich in n-3 LCPUFA (EPA or DHA) from belly oil from rainbow trout and phenolic compounds of cold-pressed maqui seed oil by enzymatic interesterification with *Thermomyces lanuginosus* lipase in supercritical CO_2_.

The tocopherol content of oils is a remarkable factor in health benefits, particularly concerning antioxidant and anti-inflammatory properties. Differences observed among oils underscored the importance of selecting and optimizing oil sources to maximize nutritional and therapeutic values. The tocopherol content of the AL shows that the blending of interesterified oils can notably impact the final tocopherol profile. This finding is crucial for developing nutritional supplements and functional foods with enhanced health benefits.

In conclusion, the enzymatic interesterification of belly oil from rainbow trout and cold-pressed maqui seed oil under supercritical CO_2_ conditions not only modified the TAG composition to produce lipids with enhanced nutritional and antioxidant properties but also optimized the process through a systematic approach. The study demonstrated that by manipulating oil ratios and operational parameters, it was possible to maximize both the yield and the health-promoting characteristics of the resultant oil, thereby rendering it suitable for functional food applications and dietary supplements. This process not only resulted in a high-quality product but also offered remarkable advantages in terms of oxidative stability and bioactivity, positioning it favorably in the realm of health-conscious consumer products.

## Figures and Tables

**Table 1:** Experimental Design Using RSM for Enzymatic Interesterification under Supercritical CO_2_ condition. Effect of the belly oil from rainbow trout/cold-pressed maqui seed oil ratio and supercritical CO_2_ temperature and pressure independent variables of the enzymatic interesterification on yield.

Experiment (No.)	Belly Oil from Rainbow Trout/Cold-Pressed Maqui Seed Oil Ratio (*w*/*w*)	Supercritical CO_2_ Temperature (°C)	Supercritical CO_2_ Pressure (Bar)	Yield (%)
1	10/90	40	200	11.0
2	90/10	40	200	28.0
3	10/90	60	200	19.0
4	90/10	60	200	28.0
5	10/90	50	100	1.0
6	90/10	50	100	1.0
7	10/90	50	300	43.0
8	90/10	50	300	63.0
9	50/50	40	100	3.0
10	50/50	60	100	43.0
11	50/50	40	300	71.0
12	50/50	60	300	64.0
13	50/50	50	200	31.0
14	50/50	50	200	52.0
15	50/50	50	200	11.1

**Table 2:** EPA and DHA (g·100 g^−1^ total FAs) content obtained by enzymatic interesterification under supercritical CO_2_ of belly oil from rainbow trout and cold-pressed maqui seed oil using RSM.

Experiment (N°)	Fatty Acid (FA) Content (g·100 g^−1^ Total FAs)		
	EPA	DHA	EPA + DHA
1	0.52 ± 0.00	0.54 ± 0.00	1.06
2	4.99 ± 0.04	4.44 ± 0.01	9.43
3	0.57 ± 0.01	0.55 ± 0.02	1.12
4	5.05 ± 0.03	4.83 ± 0.04	9.88
5	nd	nd	nd
6	nd	nd	nd
7	0.46 ± 0.00	0.43 ± 0.00	0.89
8	5.12 ± 0.01	5.26 ± 0.01	10.38
9	nd	nd	nd
10	2.71 ± 0.02	2.8 ± 0.02	5.51
11	2.79 ± 0.07	2.82 ± 0.06	5.61
12	2.75 ± 0.01	2.40 ± 0.10	5.15
13	2.44 ± 0.00	2.22 ± 0.02	4.66
14	2.76 ± 0.00	2.72 ± 0.02	5.48
15	2.36 ± 0.00	1.84 ± 0.02	4.20

nd: not determined.

**Table 3:** Tocopherol content from enzymatic interesterification under supercritical CO_2_ of belly oil from rainbow trout and cold-pressed maqui seed oil using RSM.

Experiment (No.)	Tocopherol Content of Formulations from Enzymatic Interesterification (mg·kg^−1^ Oil)				
	α-Tocopherol	α-Tocotrienol	β-Tocopherol	γ-Tocopherol	δ-Tocopherol
1	64.72 ± 28.64	2.83 ± 0.07	6.91 ± 2.28	102.27 ± 19.47	13.37 ± 3.26
2	227.66 ± 19.17	7.03 ± 1.24	1.09 ± 0.25	49.04 ± 2.65	8.43 ± 0.60
3	7.57 ± 3.06	4.74 ± 0.44	3.26 ± 0.48	47.81 ± 5.77	14.77 ± 1.07
4	0.57 ± 0.24	0.13 ± 0.05	0.14 ± 0.08	1.97 ± 0.22	1.29 ± 1.51
5	nd	nd	nd	nd	nd
6	nd	nd	nd	nd	nd
7	102.85 ± 27.89	1.52 ± 1.47	8.87 ± 0.52	154.51 ± 12.15	15.90 ± 2.40
8	97.10 ± 18.40	1.26 ± 0.21	1.33 ± 0.09	33.70 ± 0.05	6.03 ± 3.39
9	nd	nd	nd	nd	nd
10	0.15 ± 0.02	0.11 ± 0.02	0.49 ± 0.32	0.45 ± 0.27	0.67 ± 0.12
11	253.25 ± 10.38	0.77 ± 0.72	5.34 ± 0.58	93.50 ± 3.23	3.62 ± 1.10
12	30.99 ± 3.98	0.13 ± 0.08	0.36 ± 0.10	46.77 ± 2.12	4.70 ± 4.95
13	14.90 ± 3.82	0.01 ± 0.00	1.23 ± 0.14	50.27 ± 6.52	3.63 ± 0.14
14	256.09 ± 10.86	0.25 ± 0.00	0.52 ± 0.18	99.39 ± 3.95	0.17 ± 0.07
15	135.50 ± 4.52	0.13 ± 0.46	0.88 ± 0.34	74.83 ± 0.79	1.90 ± 2.45

nd: not determined.

**Table 4:** Optimization of Enzymatic Interesterification Process Variables of Belly Oil from Rainbow Trout/Cold-Pressed Maqui Seed Oil using Supercritical CO_2_ by RSM to obtain an antioxidant lipid.

Response Variables	Interesterification Process Variables			Stationary Point	Optimized Values
	Belly Oil from Rainbow Trout/Cold- Pressed Maqui Seed Oil (*w*/*w*) Ratio	Supercritical CO_2_ Temperature (°C)	Supercritical CO_2_ Pressure (Bar)		
**Part a: Optimization of the process variables**					
Yield (%)	90.0/10.0	40.0	299.69	Maximum	78.20
EPA content (g·100 g^−1^ total FAs)	90.0/10.0	60.0	299.69	Maximum	6.43
DHA content (g·100 g^−1^ total FAs)	90.0/10.0	60.0	299.69	Maximum	6.25
α-tocopherol (mg·kg^−1^ oil)	90.0/10.0	40.1	300.00	Maximum	221.20
α-tocotrienol (mg·kg^−1^ oil)	90.0/10.0	40.0	215.61	Maximum	6.52
β-tocopherol (mg·kg^−1^ oil)	10.0/90.0	40.0	286.09	Maximum	10.35
γ-tocopherol (mg·kg^−1^ oil)	10.8/80.2	40.0	300.00	Maximum	166.18
δ-tocopherol (mg·kg^−1^ oil)	10.0/90.0	40.0	299.72	Maximum	12.69
**Part b: Multiple response optimization of the response variables and desirability**					
	**Optimized process variables for obtain LA**				
Yield (%)	81.4/18.6	40.0	299.99	Maximum	77.10
EPA content (g·100 g^−1^ total FAs)				Maximum	5.12
DHA content (g·100 g^−1^ total FAs)				Maximum	4.95
α-tocopherol (mg·kg^−1^ oil)				Maximum	217.96
α-tocotrienol (mg·kg^−1^ oil)				Maximum	4.28
β-tocopherol (mg·kg^−1^ oil)				Maximum	3.48
γ-tocopherol (mg·kg^−1^ oil)				Maximum	64.48
δ-tocopherol (mg·kg^−1^ oil)				Maximum	6.39
**Part c: Experimental validation of the multiple response optimization of the response variables of Part b**					
**Optimized process variables by RSM**					
Yield (%)	81.4/18.6	40.0	299.99	Maximum	78.80
EPA content (g·100 g^−1^ total FAs)				Maximum	4.59
DHA content (g·100 g^−1^ total FAs)				Maximum	4.03
α-tocopherol (mg·kg^−1^ oil)				Maximum	101.76
α-tocotrienol (mg·kg^−1^ oil)				Maximum	0.00
β-tocopherol (mg·kg^−1^ oil)				Maximum	5.25
γ-tocopherol (mg·kg^−1^ oil)				Maximum	47.84
δ-tocopherol (mg·kg^−1^ oil)				Maximum	7.11

**Table 5:** Color L*, a*, b* parameters for cold-pressed maqui seed oil, belly oil from rainbow trout and the antioxidant lipid *.

Oil	L*	a*	b*
Cold-pressed maqui seed oil	92.1 ± 0.0 ^a^	−12.5 ± 0.0 ^c^	51.8 ± 0.0 ^b^
Belly oil from rainbow trout	75.3 ± 0.0 ^c^	32.3 ± 0.0 ^a^	93.2 ± 0.0 ^a^
Antioxidant lipid	78.4 ± 0.0 ^b^	14.2 ± 0.0 ^b^	48.3 ± 0.0 ^c^

* Values are expressed as mean ± standard deviation (SD) (*n* = 3). In each column, values with different superscript letters (^a,b,c^) indicate significant differences (*p* < 0.05) according to Fisher’s test.

**Table 6:** Temperatures of peaks (T_Peak_), onset temperature (T_Onset_), endset temperature (T_Endset_), and ∆H (J·g^−1^) from the melting curves of cold-pressed maqui seed oil, belly oil from rainbow trout and antioxidant lipid *.

Oil	ΔH (J·g^−1^)	T_Onset_ (°C)	T_Peak1_ (°C)	T_Peak2_ (°C)	T_Peak3_ (°C)	T_Peak4_ (°C)	T_Endset_ (°C)
Cold- pressed Seed Maqui	214.38 ± 34.44 ^a^	−77.47 ± 2.28 ^a^	−73.70 ± 2.028 ^a^	−33.74 ± 0.53 ^a^	−22.72 ± 0.43 ^b^	-	58.07 ± 0.00 ^a^
Belly	196.82 ± 8.02 ^a^	−74.52 ± 0.32 ^a^	−70.62 ± 0.30 ^a^	−42.32 ± 1.24 ^b^	−19.06 ± 0.24 ^a^	−4.12 ± 1.01 ^b^	58.06 ± 0.00 ^a^
Antioxidant lipid	205.03 ± 2.17 ^a^	−74.94 ± 2.52 ^a^	−71.76 ± 0.83 ^a^	−47.39 ± 1.47 ^c^	−26.05 ± 0.47 ^c^	1.61 ± 0.06 ^a^	58.07 ± 0.00 ^a^

* Values are expressed as mean ± standard deviation (SD) (*n* = 3). In each column, values with different superscript letters (^a,b,c^) indicate significant differences (*p* < 0.05) according to Fisher’s test.

**Table 7:** Chemical analyses of cold-pressed maqui seed oil, belly oil from rainbow trout, and antioxidant lipid *.

Oil	Free Acidity (% Oleic Acid)	Peroxide Value (mEq O_2_·kg^−1^ Oil)	*p*- Anisidine Value	Conjugated Dienes	Conjugated Trienes	TOTOX Value
Cold- pressed maqui Seed	0.22 ± 0.04 ^a^	0.23 ± 0.06 ^b^	9.12 ± 0.00 ^a^	0.05 ± 0.00 ^a^	0.02 ± 0.00 ^a^	9.58 ± 0.12 ^a^
Belly from rainbow trout	0.16 ± 0.02 ^b^	0.31 ± 0.03 ^b^	6.86 ± 0.03 ^b^	0.06 ± 0.02 ^a^	0.02 ± 0.00 ^a^	7.49 ± 0.06 ^b^
Antioxidant lipid	0.11 ± 0.03 ^b^	0.71 ± 0.07 ^ba^	0.16 ± 0.06 ^c^	0.00 ± 0.00 ^b^	0.00 ± 0.00 ^b^	1.57 ± 0.20 ^c^

* Values are expressed as mean ± standard deviation (SD) (*n* = 3). In each column, values with different superscript letters (^a,b,c,ba^) indicate significant differences (*p* < 0.05) according to Fisher’s test.

**Table 8:** Composition and quantification of fatty acids (FA) (g·100 g^−1^ total FAs) of cold-pressed maqui seed oil, belly oil from rainbow trout and antioxidant lipid *.

Systematic Name	Abbreviation Name	Oil		
		Cold-Pressed Maqui Seed Oil	Belly Oil from Rainbow Trout	Antioxidant Lipid
Lauric acid	C12:0	nd	0.07 ± 0.00 ^b^	0.10 ± 0.02 ^a^
Myristic acid	C14:0	0.14 ± 0.00 ^c^	2.58 ± 0.02 ^b^	3.06 ± 0.17 ^a^
Pentadecanoic acid	C15:0	nd	0.19 ± 0.00 ^a^	0.19 ± 0.06 ^a^
Palmitic acid	C16:0	8.52 ± 0.01 ^c^	12.34 ± 0.05 ^b^	13.69 ± 0.23 ^a^
Palmitelaidic acid	C16:1n-7t	0.14 ± 0.00 ^c^	0.18 ± 0.00 ^a^	0.16 ± 0.04 ^b^
Palmitoleic acid	C16:1n-7	nd	4.19 ± 0.02 ^a^	4.22 ± 0.11 ^a^
Heptadecanoic acid	C17:0	0.07 ± 0.00 ^c^	0.18 ± 0.00 ^a^	0.15 ± 0.08 ^b^
Heptadecenoic acid	C17:1n-7	nd	0.37 ± 0.00 ^a^	0.27 ± 0.15 ^b^
Stearic acid	C18:0	5.35 ± 0.00 ^a^	3.66 ± 0.02 ^b^	3.56 ± 0.07 ^b^
Oleic acid	C18:1n-9	32.78 ± 0.01 ^a^	19.78 ± 0.12 ^c^	25.27 ± 0.04 ^b^
Cis-Vaccenic acid	C18:1n-7	1.15 ± 0.00 ^b^	2.40 ± 0.01 ^a^	2.31 ± 0.04 ^a^
Linoleic acid	C18:2n-6	30.98 ± 0.00 ^a^	16.25 ± 0.08 ^b^	29.43 ± 0.17 ^a^
Arachidic acid	C20:0	0.36 ± 0.00 ^a^	nd	0.003 ± 0.002 ^b^
Gamma linolenic acid	C18:3n-6	0.25 ± 0.00 ^a^	nd	0.10 ± 0.02 ^b^
8-Eicosanoic acid	C20:1n-12	nd	0.53 ± 0.00 ^a^	0.19 ± 0.02 ^b^
α-Linolenic acid	C18:3n-3	19.78 ± 0.02 ^a^	2.50 ± 0.02 ^c^	5.56 ± 0.10 ^b^
11-Eicosenoic acid	C20:1n-9	nd	1.47 ± 0.01 ^a^	0.01 ± 0.02 ^b^
Eicosadienoic acid	C20:2n-6	nd	0.66 ± 0.00 ^b^	0.91 ± 0.01 ^a^
Arachidonic acid	C20:4n-6	nd	1.06 ± 0.04 ^a^	0.34 ± 0.03^b^
Docosadienoic acid	C22:2n-6	nd	0.64 ± 0.00 ^a^	0.10 ± 0.02 ^b^
Eicosapentaenoic acid (EPA)	C20:5n-3	nd	5.26 ± 0.04 ^a^	4.59 ± 0.07 ^b^
Lignoceric acid	C24:0	0.28 ± 0.0 ^a^	nd	0.01 ± 0.01 ^a^
Docosapentaenoic acid	C22:5n-3	nd	2.12 ± 0.01 ^a^	1.77 ± 0.06 ^b^
Docosahexaenoic acid (DHA)	C22:6n-3	nd	5.23 ± 0.05 ^a^	4.03 ± 0.13 ^b^
Total saturated fatty acids		14.72 ^c^	19.02 ^b^	20.77 ^a^
Total monounsaturated fatty acids		34.07 ^a^	28.92^c^	32.43 ^b^
Total polyunsaturated fatty acids		51.01 ^a^	33.72 ^c^	46.83 ^b^
Total n-3 polyunsaturated fatty acids		19.78 ^a^	15.11 ^b^	15.95 ^c^
EPA + DHA		nd	10.49 ± 0.09 ^a^	8.62 ± 0.17 ^b^

* Values are expressed as mean ± standard deviation (SD) (*n* = 3). In each row, values with different superscript letters (^a,b,c^) indicate significant differences (*p* < 0.05) according to Fisher’s test. nd: not determined. The antioxidant lipid independent variables were: 81.4/18.6 (*w*/*w*) for belly oil from rainbow trout/cold-pressed maqui seed oil ratio (*w*/*w*), 40.0 °C, and 299.9 bar.

**Table 9:** Total phenolic content value of the oil obtained from cold-pressed maqui seed oil, belly oil, and antioxidant lipid *.

Oil	Total Phenols (mg GAE·g^−1^ Oil)
Cold-pressed seed Maqui	3.27 ± 0.15 ^a^
Belly from rainbow trout	0.00 ± 0.00 ^c^
Antioxidant lipid	1.09 ± 0.06 ^b^

* Values are expressed as mean ± standard deviation (SD) (*n* = 3). In each column, values with different superscript letters (^a,b,c^) indicate significant differences (*p* < 0.05) according to Fisher’s test.

**Table 10:** Tocopherol concentration (mg·kg^−1^ oil) in cold-pressed maqui seed oil, belly oil from rainbow trout, and the antioxidant lipid *.

Oil	Concentration (mg·kg^−1^ Oil)				
	α-Tocopherol	α-Tocotrienol	β-Tocopherol	γ-Tocopherol	δ-Tocopherol
Cold-pressed Maqui seed	339.09 ± 5.15 ^a^	0.91 ± 0.27 ^a^	11.85 ± 0.73 ^a^	135.52 ± 38.03 ^a^	3.96 ± 0.22 ^a^
Belly from rainbow trout	191.80 ± 1.71 ^b^	2.92 ± 0.43 ^b^	0.20 ± 0.18 ^b^	18.01 ± 0.40 ^b^	1.40 ± 0.21 ^b^
Antioxidant lipid (AL)	101.76 ± 0.05 ^c^	0.00 ± 0.05 ^a^	5.25 ± 0.09 ^c^	47.84 ± 0.22 ^c^	7.11 ± 1.51 ^b^

* Values are expressed as mean ± standard deviation (SD) (*n* = 3). In each column, values with different superscript letters (^a,b,c^) indicate significant differences (*p* < 0.05) according to Fisher’s test.

## Data Availability

All the data are contained within the manuscript and the Supplementary Materials.

## Supplementary Materials

The following supporting information can be downloaded at: https://www.mdpi.com/article/10.3390/md22120547/s1, Table S1: ANOVA and regression coefficients of the first-order (Y_2_, Y_3_, Y_4_, Y_6_ and Y_8_) and second-order (Y_1_, Y_5_, and Y_7_) polynomial models and *p* values for the different response variables, i.e., oil yield (%), EPA and DHA (g·100 g^−1^ total FAs) and tocopherol concentration (mg·kg^−1^ oil) obtained from the interesterification of belly oil from rainbow trout and cold-pressed maqui seed oil under supercritical CO_2_*.
