# Supplementary material for: Enzymatic Interesterification of Cold-Pressed Maqui (Aristotelia chilensis (Mol.) Stuntz) Seed Oil and Belly Oil from Rainbow Trout (Oncorhynchus mykiss) Through Supercritical CO2
Source: Mar Drugs. 2024 Dec 4;22(12):547. doi: 10.3390/md22120547 (PMC11678877; doi:10.3390/md22120547)
Supplement: Supplementary file 1 [file marinedrugs-22-00547-s001.zip › marinedrugs-3229279-supplementary.pdf]

Table S1-Supplementary. ANOVA and regression coefficients of the first-order (Y_2, Y_3, Y_4, Y_6 and Y_8) and second-order (Y_1, Y_5, and Y_7) polynomial models and *p* values for the different response variables, i.e., oil yield (%), EPA and DHA (g·100 g^{-1} total FAs) and tocopherol concentration (mg·kg^{-1} oil) obtained from the interesterification of belly oil from rainbow trout and cold-pressed maqui seed oil under supercritical CO_2*.

**Process Variables** **	**Responses Variables**															
	***Y*₁ (Oil Yield)**		***Y*₂ (EPA)**		***Y*₃ (DHA)**		***Y*₄ (α-Tocopherol)**		***Y*₅ (α-Tocotrienol)**		***Y*₆ (β-Tocopherol)**		***Y*₇ (γ-Tocopherol)**		***Y*₈ (δ-Tocopherol)**	
	**Coefficient**	***p* value**	**Coefficient**	***p* value**	**Coefficient**	***p* value**	**Coefficient**	***p* value**	**Coefficient**	***p* value**	**Coefficient**	***p* value**	**Coefficient**	***p* value**	**Coefficient**	***p* value**
Constant	-264.73		-0.89		-0.72		251.42		25.30		-10.48		19.53		4.48	
Linear																
A	0.14	0.28	-0.02	0.00	-0.02	0.00	0.47	0.51	0.15	0.74	-0.05	0.00	0.82	0.01	-0.09	0.05
B	5.02	0.69	0.03	0.36	0.03	0.33	-6.33	0.04	-1.35	0.02	0.16	0.01	-1.85	0.05	-0.05	0.76
C	1.48	0.01	0.00	0.02	0.00	0.01	0.60	0.05	0.06	0.10	0.11	0.00	0.79	0.00	0.04	0.04
Quadratic																
A × A	-	-	-	-	-	-	-	-	0.00	0.00	0.00	0.01	-	-	-	-
B × B	-	-	-	-	-	-	-	-	0.02	0.00	-	-	-	-	-	-
C × C	-	-	-	-	-	-	-	-	0.00	0.00	-	-	-	-	-	-
Interaction																
A × B	-	-	-	-	-	-	-	-	0.00	0.00	-	-	-	-	-	-
A × C	-	-	0.00	0.04	0.00	0.03	-	-	-	-	0.00	0.00	-0.01	0.02	-	-
B × C	-0.03	0.00	-	-	-	-	-	-	-	-	0.00	0.02	-	-	-	-
R^2	72.86		78.76		79.88		49.08		94.77		94.20		83.07		47.88	
Adjusted R^2	62.01		70.26		71.84		35.19		89.54		89.85		76.30		33.66	
SE	14.08		1.02		0.96		77.79		0.67		0.89		22.96		4.57	
MAE	9.39		0.68		0.65		53.82		0.38		0.59		13.68		3.32	
DW value	1.62	0.39	1.97	0.65	2.10	0.74	1.87	0.53	2.35	0.50	1.56	0.28	1.42	0.25	0.85	0.02
Lag 1 RA	0.08		0.00		-0.07		0.02		-0.23		0.13		0.23		0.51	
Lack of Fit	1146.87	0.89	10.27	0.03	8.74	0.16	37478.6	0.92	3.14	0.02	6.11	0.12	4065.07	0.65	224.107	0.11
Pure Error	836.61		0.09		0.39		29088.1		0.03		0.25		1206.32		6.00	

* Abbreviations: R^2 (regression coefficient), SE (standard error), MAE (mean absolute error), DW (Durbin–Watson), and RA (residual autocorrelation). ** Process variables: A (belly oil from rainbow trout/ cold-pressed maqui seed oil, w/w), B (temperature, °C), and C (pressure, bar). Responses variables: (Y_1) yield, (Y_2) EPA, (Y_3) DHA and ($Y_{4–8}$) tocopherol concentration.
